# The risk of antidepressant-induced hyponatremia: A meta-analysis of antidepressant classes and compounds

**DOI:** 10.1192/j.eurpsy.2024.11

**Published:** 2024-02-26

**Authors:** Tim Gheysens, Filip Van Den Eede, Livia De Picker

**Affiliations:** 1 Collaborative Antwerp Psychiatric Research Institute, Faculty of Medicine and Health Sciences, University of Antwerp, Antwerp, Belgium; 2 Scientific Initiative of Neuropsychiatric and Psychopharmacological Studies, University Psychiatric Centre Campus Duffel, Duffel, Belgium; 3 Department of Psychiatry, Antwerp University Hospital, Edegem (Antwerp), Belgium

**Keywords:** adverse drug events, antidepressive agents, hyponatremia, inappropriate ADH syndrome, psychiatry

## Abstract

**Background:**

Hyponatremia (hypoNa) is a potentially serious adverse event of antidepressant treatment. Previous research suggests the risk of drug-induced hyponatremia differs between antidepressants. This meta-analysis sought to determine the risk of antidepressant-induced hypoNa, stratified by different compounds and classes.

**Methods:**

A PRISMA-compliant systematic search of Web of Science and PubMed databases was performed from inception until Jan 5, 2023, for original studies reporting incidences or risks of hypoNa in adults using antidepressants. We modelled random-effects meta-analyses to compute overall event rates and odds ratios of any and clinically relevant hypoNa for each compound and class, and ran head-to-head comparisons based on hypoNa event rates. We conducted subgroup analyses for geriatric populations and sodium cut-off value. The study is registered with PROSPERO, CRD42021269801.

**Results:**

We included 39 studies (n = 8,175,111). Exposure to antidepressants was associated with significantly increased odds of hypoNa (k = 7 studies, OR = 3.160 (95%CI 1.911-5.225)). The highest event rates were found for SNRIs (7.44%), SSRIs (5.59%), and TCAs (2.66%); the lowest for mirtazapine (1.02%) and trazodone (0.89%). Compared to SSRIs, SNRIs were significantly more likely (k = 10, OR = 1.292 (1.120 – 1.491), p < 0.001) and mirtazapine significantly less likely (k = 9, OR = 0.607 (0.385 – 0.957), p = 0.032) to be associated with hypoNa.

**Conclusion:**

Our meta-analysis demonstrated that, while no antidepressant can be considered completely risk-free, for hypoNa-prone patients mirtazapine should be considered the treatment of choice and SNRIs should be prescribed more cautiously than SSRIs and TCAs.

## Introduction

Hyponatremia (hypoNa) is considered an important adverse event of antidepressant therapy because of its potentially serious impact on a patient’s general health [1]. In hospitalized patients, and irrespective of whether it was community- or hospital-acquired or hospital-aggravated, hypoNa is associated with an increased risk of mortality, longer hospital stays, and eventual referral to a long-term care facility [2].

In laboratory investigations, hypoNa is commonly defined as a serum sodium concentration <135 mmol/L, where values of 130–134 mmol/L are classified as mild, 125–129 mmol/L as moderate, and <125 mmol/L as severe hypoNa. Symptoms may include nausea and vomiting, headache, confusion, fatigue, irritability, and muscle weakness or spasms up to the most serious complications such as seizures, coma, and respiratory arrest. Clinically relevant hypoNa mainly occurs when hypoNa is moderate (125–129 mmol/L) or severe (<125 mmol/L), although mild hypoNa can also be symptomatic. Given the potential for serious complications, early recognition of drug-induced hypoNa can be of vital importance. Awareness and routine screening for drug-induced hypoNa is therefore warranted in all high-risk situations.

In a previous systematic review covering the scientific literature published until 2013, we found evidence supporting the hypothesis that the risk of drug-induced hypoNa differs for antidepressants with different pharmacodynamic profiles. We noted the highest risk for selective serotonin reuptake inhibitors (SSRIs) and serotonin-norepinephrine reuptake inhibitors (SNRIs), with a more attenuated risk for tricyclic antidepressants (TCAs) and some atypical compounds [3]. Other reviews drew similar conclusions regarding the relatively higher risk of hypoNa for SSRIs and SNRIs in geriatric populations, although they were not able to quantitatively determine differences in the risk profiles of the two classes [1, 4, 5]. In their review, Greenblatt et al. concluded, however, that the evidence for non-SSRI antidepressants having a lower risk of hypoNa is still very limited and that, given the elevated risk of hypoNa in older patients, well-tolerated alternatives to SSRIs should be evaluated [6]. Candidate antidepressant(s) suggested vary from TCA and trazodone to mirtazapine or bupropion [3, 5, 6], but a research synthesis of their hypoNa risks or quantitative comparisons of antidepressant classes or compounds are still lacking.

The current report not only updates our previous systematic review with the literature published in the past decade, but, most importantly, it also comprises a quantitative meta-analysis of the evidence to date, describing the overall event rate and risk of both any and clinically relevant hypoNa (see Section 2.3) stratified by antidepressant compound and class.

## Methods

We performed a meta-analysis of studies reporting on the event rate or risk of hypoNa in persons using antidepressants (PROSPERO ID: CRD42021269801; an overview of updates of our protocol is available in the Supplementary material). The study was conducted according to the 2020 PRISMA (Preferred Reporting Items for Systematic Reviews and Meta-analysis) guidelines [7].

### Search strategy

We conducted an independent and systematic multi-step search to identify all eligible articles published from inception to January 5, 2023, in PubMed and Web of Science. We used the same search strings as in our previous work, with the addition of (es)ketamine and the names of individual SNRIs and atypical antidepressants. The full search strings can be found in the Supplementary material.

After the removal of duplicates, the first author (TG) screened all titles and abstracts to select potentially relevant studies, after which two authors (TG, FVDE) independently completed the study selection, deciding whether – based on the titles, abstracts, and full texts of all individual studies – they fulfilled the eligibility criteria. Disagreements were resolved by consulting with the third author (LDP).

### Eligibility criteria

Our inclusion criteria were original studies published in peer-reviewed journals reporting on adults exposed to drugs classified as antidepressants (N06A) according to the 2023 ATC/DDD Index that reported event rates or association measures (OR, risk ratio, hazard ratio, or associated metrics) for hypoNa.

Our exclusion criteria were (1) studies not including human participants; (2) studies not reporting incidences or association measures for hypoNa in participants exposed to antidepressants; (3) non-peer-reviewed studies, reviews, clinical case reports, abstracts, conference proceedings, or preprints, and (4) duplicate publications. Searches and eligibility screenings were not limited to any particular language or time period.

### Outcome measures

The primary outcome was the risk of hypoNa (i.e., any documented case of hyponatremia or serum sodium <135 mmol/L). Secondary outcomes were clinically relevant hypoNa (i.e., symptomatic hypoNa, hypoNa requiring treatment or hospitalization, or serum sodium <130 mmol/L). Risks were assessed through event rates in cohort studies and through crude and adjusted odds ratios (ORs, aORs) with related 95% CIs in case–control studies. While ORs provide information on the observed outcomes, aORs provide insight into the direct risks after controlling for potential confounders such as age, sex, and comedication. An OR and its 95% CIs >1 indicate an increased likelihood of hypoNa in subjects exposed to the culprit drug compared to unexposed subjects. For the longitudinal cohort study of Coupland and colleagues, we estimated adjusted 1-year event rates from the absolute risk of hyponatremia at 1 year from baseline, adjusted for confounders, the number of events over the total follow-up period, and the average follow-up time to increase comparability with other studies (see Supplementary Table S5) [28]. For each outcome, we considered both class- and compound-specific exposure variables: SSRIs (N06AB), TCAs (N06AA), MAOIs (N06AF & N06AG). We split the N06AX category into SNRIs and other (atypical) antidepressants (see Supplementary Table S3 for details). We report compound-specific outcomes when at least two studies were available.

### Data extraction and processing

For each included study, meta-analytical data was extracted by the first author (TG) and independently verified by two other authors (FVDE, LDP). A full list of the extracted variables is available in the Supplementary material.

For case–control studies, crude ORs were calculated using outcomes and sample sizes for groups of interest. We assumed study participants were only exposed to a single antidepressant during the study period for which event rates were given. When outcomes were reported for a heterogeneous mixture of antidepressant classes or compounds, we contacted the authors to request outcome data stratified by compound. This was the case for two studies [8, 9]. For one of these two studies, the authors responded so we could include unpublished data from the study conducted by Giorlando et al. [9]. In the case–control study by Movig et al., the frequency data for individual antidepressants in two heterogeneous (serotonergic and non-serotonergic) antidepressant groups were given, but stratification by individual compound was not feasible due to the small sample size [10].

We used raw data as input for the calculation of event rates and crude ORs, and aOR with their 95% CI as input for the calculation of aORs. If the confidence interval provided for aOR was too asymmetric for analysis, we widened it until it met the symmetry threshold (fudge ratio 2.0). Details about which data was adapted can be found in Supplementary Table S5.

### Study quality

Author TG assessed the quality of all eligible studies using the Newcastle-Ottawa Scale (NOS) that comprises eight items categorized into three groups: the selection of study groups; the comparability of groups; and the ascertainment of the outcome of interest [11]. A higher score indicates higher methodological quality.

### Data synthesis and analysis

For both primary and secondary outcomes, we used DerSimonian and Laird random-effects models expecting high heterogeneity, with percent residual variation being indexed by Higgins’ I² statistic (I² = 25–49%: low; I² = 50–74%: moderate; I² ≥ 75%: high) and uncertainty measured by 95% CIs. For each outcome, the primary meta-analysis describes the event rates and ORs across all compounds, while the secondary analysis does so for each antidepressant class, including a head-to-head comparison of different antidepressant classes or compounds, with SSRI as the reference class. Findings are presented in forest plots. Publication bias was evaluated through visual inspection of a funnel plot displaying the estimates against their standard errors. If at least 10 studies were included in the meta-analysis, the Egger linear regression test was used to assess the symmetry of the effects and to statistically test for publication bias. Significance of all associations was evaluated with p-values (with alpha set to <0.05).

For all outcome measures, we tested the effect of the following covariates in subgroup analyses or meta-regressions (if k studies ≥10; method of moments with Knapp-Hartung adjustment): (1) degree of hyponatremia (serum sodium <135 mmol/L versus <130 mmol/L or clinical case definition); (2) age group of study population (general adult versus geriatric population, geriatric defined as a study population over 60 years of age); (3) study context (study outcomes based on electronic health records (EHRs), pharmacovigilance studies, or single or multicenter cohort studies; (4) study quality score.

All analyses were performed in Comprehensive Meta-Analysis version 4.0.000 (2022, Biostat, Inc.).

## Results

### Study selection

**Figure 1.**
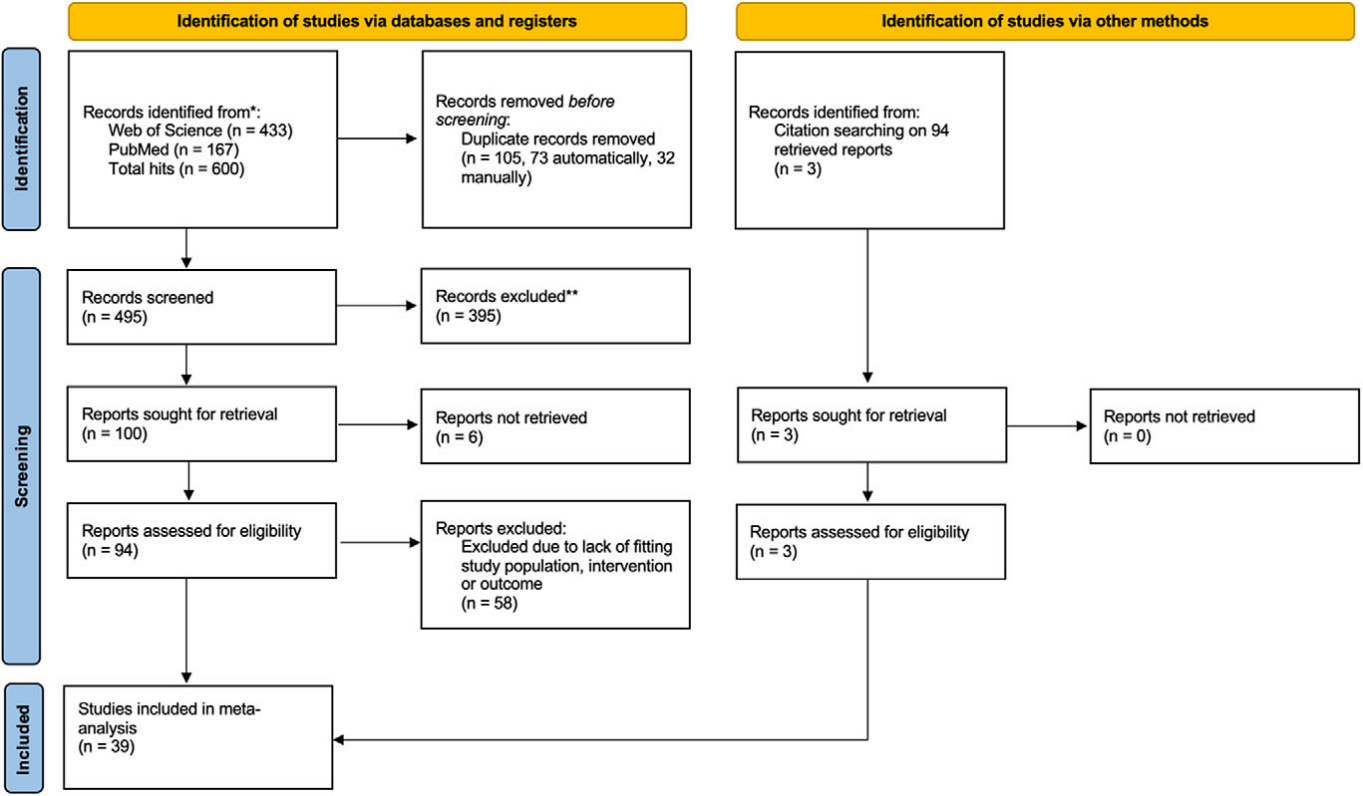
PRISMA flowchart of study inclusion process.

The systematic search yielded 600 hits, of which 94 concerned original studies; of these 36 met the eligibility criteria, while 3 additional studies were included based on references (see the PRISMA flowchart in Figure 1). The total sample for meta-analysis thus included 39 studies with data on 8,175,111 individuals using antidepressants and 115,553 cases of hypoNa. In the total sample, the average age was 51.1 years and 61.5% of individuals were female.

An overview of the included studies is given in Table 1. Of the 39 included studies, 32 reported event rate outcomes and 9 reported OR outcomes (Kirby et al. [12] and Leth-Møller et al. [13] reported both event rates and ORs).

**Table 1.** Included studies by study type

Type of study	References	Year	Country	NOS rating (stars)	Sample size (cases/controls or number of exposed participants)	Definition or cut-off hyponatremia	Clinical outcome of hyponatremia	Mean age	Female study participants	In- or outpatients	Geriatric	SSRI	SNRI	TCA	MAOI	Atypical agents
Case–control studies	Siegler et al. [31]	1995	USA	7	64/192	<130 mmol/L	Asymptomatic	62.3/43.4	79.7%/65.6%	In	No	27%	/	60%	13%	
	Kirby et al. [12]	2002	Australia	5	60/125	<135 mmol/L	Mixed	74.2	65%	In	Yes	81%	19%	/	/	/
	Movig et al. [10]	2002	The Netherlands	7	203/608	ICD-code	Hospitalization	71/71	73.4%/73.4%	In	No	YES	YES	YES	/	/
	Leth-Møller et al. [13]	2016	Denmark	6	72,509/638,352	<135 mmol/L	Not given	See study^a^	See study^a^	Out	No	YES	YES	YES	/	YES
	Rochoy et al. [32]	2018	France (BNPV)	3	3,397/252,373	MedDRA	Not given	Not given	Not given	Not given	No	50.5%	19%	13.5%	0.5%	17%
	Farmand et al. [33]	2018	Sweden	6	11,213/44,801	ICD-code	Hospitalization	76/76	72%/72%	In	No	65%	5%	9%	/	21%
	Mazhar et al. [23]	2019	USA (FAERS)	5	13,307/2,771,233	MedDRA	Not given	61.5/49.1	57.3%/55.6%	Not given	No	47%	24%	7.4%	0.1%	21.5%
	Jun et al. [34]	2021	Korea	4	913/18,260	treatment with hypertonic saline or tolvaptan	Hospitalization	Not given	52.1%/52.1%	In	Yes	40%	13%	38%	/	9%
	Mannheimer et al. [35]	2021	Sweden	7	11,213/44,801	ICD-code	Hospitalization	76/76	72%/72%	In	No	100%	/	/	/	/
Prospective cohort studies	Huyse et al. [28]	1994	The Netherlands	2	37	clinician-based	Symptomatic	60	50%	In	No	100%	/	/	/	/
	Spigset et al. [36]	1997	Sweden	5	8	<135 mmol/L	Not given	29	0%	Out	No	100%	/	/	/	/
	Fabian et al. [37]	2003	USA	5	15	<135 mmol/L	Not given	75.7	46.7%	Out	Yes	100%	/	/	/	/
	Fabian et al. [38]	2004	USA	7	75	<135 mmol/L	Mixed	75.3	70.7%	Out	Yes	100%	/	/	/	/
	Rowbotham et al. [39]	2005	USA	3	47	Clinician-based	Hospitalization	72	57.4%	Out	No	32%	/	68%	/	/
	Roxanas et al. [18]	2007	Australia	5	58	<130 mmol/L	Not given	76	90%	In and out	Yes	/	100%	/	/	/
	Shakibaei et al. [40]	2010	Iran	2	126	<135 mmol/L	Symptomatic	Not given	Not given	In	No	100%	/	/	/	/
	Shetty et al. [30]	2015	India	7	74	<135 mmol/L	Not given	Not given	56.8%	In and out	No	YES	YES	YES	/	YES
	EFFECTS trial collaboration [41]	2020	Sweden	6	750	<130 mmol/L	Not given	70.6	38%	In	No	100%	/	/	/	/
	Sarkar et al. [20]	2021	India	4	200	<135 mmol/L	Mixed	47.6	See study^a^	Out	No	100%	/	/	/	/
Retrospective cohort studies	Brymer et al. [42]	1992	USA	3	40	<135 mmol/L	Not given	Not given	Not given	In	Yes	62.5%	/	37.5%	/	/
	Pillans et al. [19]	1994	New Zealand	1	5,628	clinician’s report	Mixed	Not given	Not given	Not given	No	100%	/	/	/	/
	Bouman et al. [43]	1998	UK	2	32	<135 mmol/L	Mixed	76.1	46.9%	In	Yes	100%	/	/	/	/
	Strachan et al. [44]	1998	Australia / New Zealand	3	55	<135 mmol/L	Mixed	74.3	60%	In	Yes	100%	/	/	/	/
	Wilkinson et al. [45]	1999	New Zealand	3	845	<130 mmol/L	Not given	Not given	Not given	In and out	Yes	100%	/	/	/	/
	Spigset et al. [24]	1999	Sweden	3	1,202	Clinician’s report	Not given	49	67.3%	Not given	No	100%	/	/	/	/
	Kirby et al. [12]	2002	Australia	5	60/125	<135 mmol/L	mixed	74.2	65%	In	Yes	81%	19%	/	/	/
	Wee et al. [25]	2004	Australia	4	103	<135 mmol/L	Not given	78.4	64%	In	Yes	100%	/	/	/	/
	Jung et al. [46]	2011	Korea	4	240	<135 mmol/L	Asymptomatic	51.5	67.1%	In	No	39%	29.5%	/	/	31.5%
	Coupland et al. [29]	2011	UK	8	60,405	ICD-code	Not given	Not given	70.1%	Out	Yes	YES	YES	YES	/	YES
	Montastruc et al. [47]	2012	France (CRPV-MP)	3	177	MedDRA	Not given	61	68.4%	Not given	No	YES	YES	/	/	/
	Giorlando et al. [9]	2013	Australia	4	131	<135 mmol/L	Not given	See study^a^	Not given	In	Yes	38%	23%	7%	/	7%
	Lange-Asschenfeldt et al. [26]	2013	Germany	3	2,606	<135 mmol/L	Mixed	Not given	Not given	In	No	25%	16.50%	20%	/	38.5%
	Leth-Møller et al. [13]	2016	Denmark	7	98,976	<3135 mmol/L	Not given	See study^a^	See study^a^	Out	No	YES	YES	YES	/	YES
	Noohi et al. [8]	2016	Canada	4	69	<135 mmol/L	Not given	See study^a^	See study^a^	In	No	YES	?	?	?	?
	Gandhi et al. [48]	2017	Canada	7	138,246	ICD-code	Hospitalization	76	67.5%	Out	Yes	91%	/	/	/	9%
	Albrecht et al. [49]	2018	USA	6	15,733	ICD-code	Not given	79.7	68%	In and out	Yes	78.4%	16.4%	11.3%	/	/
	Grattagliano et al. [27]	2018	Italy	3	172	<135 mmol/L	Not given	Not given	Not given	Out	Yes	100%	/	/	/	/
	Revol et al. [50]	2018	France (BNPV)	3	3,749,800	MedDRA	Not given	Not given	65.9%	Not given	No	YES	YES	/	/	/
	Seifert et al. [51]	2021	Germany, Switzerland, Austria (AMSP)	3	243,588	<130 mmol/L	Not given	Not given	Not given	In	No	YES	YES	YES	YES	YES
	Shysh et al. [52]	2021	Canada	4	19,679	<135 mmol/L	Not given	55.5	65.3%	Out	No	100%	/	/	/	/
	Nagashima et al. [15]	2022	Japan (JADER)	7	8,473	MedDRA	Not given	Not given	Not given	Not given	Yes	54%	32%	/	/	14%

*Note*: The outcome measures of the case–control studies and cohort studies were association measures (odds ratios, OR) or event rates (number of cases of hyponatremia or number of exposed participants), respectively. The study by Leth-Møller et al. was included both as a case–control study and as a retrospective cohort study. For pharmacovigilance studies the database acronyms are mentioned in the country column (BNPV: la base nationale française de pharmacovigilance; FAERS: US Food and Drug Administration (FDA) Adverse Event Reporting System; CRPV-MP: Centre Régional de PharmacoVigilance, Midi-Pyrénées; AMSP: Arzneimittelsicherheit in der Psychiatrie; JADER: Japanese Adverse Drug Event Report). An overview of all databases or datasets used in the different studies can be found in the Supplementary material. The quality of studies was assessed using the Newcastle-Ottawa Scale (NOS). Outside of cut-off values for serum sodium concentration hyponatremia was sometimes defined by a specific coding system (ICD: International Classification of Diseases; MedDRA: Medical Dictionary for Regulatory Activities). If reported, for case–control studies the mean age and percentage of female participants is given as cases/controls and for cohort studies as one value for all exposed participants. For the case–control study by Kirby et al. these demographic values are given for the whole study population (not specific for cases and controls).

a If mean ages or female participant ratio was only retrievable for a subgroup of participants, we referred to the respective study. An overview of included antidepressant classes per study is given and specified with a percentage if the exposure to a specific antidepressant class on the total antidepressant exposure could be retrieved.

### HypoNa event rate and OR across all compounds

For all individuals exposed to antidepressants and across all compounds, we found an estimated hypoNa event rate of 4.65% (k = 32 studies, event rate = 0.0465 [95%CI 0.0248–0.0858], I^2^ = 99.88%; Supplementary Figure S6). The event rate of clinically relevant hypoNa was lower at 1.10% (k = 16, event rate = 0.0110 [0.0028–0.0421], I^2^ = 90.91; Supplementary Figure S11). Exposure to antidepressants was associated with significantly increased odds of hypoNa (k = 7, OR = 3.160 [1.911–5.225], I^2^ = 99.52%; Supplementary Figure S26) and clinically relevant hypoNa (k = 6, OR = 2.930 [1.715–5.008], I^2^ = 99.60%; Supplementary Figure S32). This increased risk remained robust for ORs adjusted for confounders like age, sex, and other drugs known to cause hyponatremia (any hypoNa k = 6, aOR = 1.891 [1.573–2.270], p < 0.001, I^2^ = 91.15%; clinically relevant hypoNa k = 6, aOR = 2.123 [1.363–3.307], p < 0.001, I^2^ = 91.76%; Supplementary Figures S36 and S43). Between-study heterogeneity was high in all analyses. Table 2 presents an overview of the different outcomes.

**Table 2.** Pooled incidences (event rates) and odds ratios (OR and aOR) for antidepressant classes

Antidepressant (class)	Number of studies	Event rate or OR (95% confidence interval)	*p*-value	I^2^ (%)
Incidence (event rate) for any hyponatremia				
Antidepressants (overall)	32	0.0465 (0.0248–0.0858)	<0.001	99.88
SSRIs	28	0.0559 (0.0289–0.1055)	<0.001	99.69
SNRIs	10	0.0744 (0.0225–0.2195)	<0.001	99.32
TCAs	8	0.0266 (0.0055–0.1184)	<0.001	98.49
Atypical antidepressants (mirtazapine only)	8	0.0102 (0.0025–0.0402)	<0.001	97.98
Risk (OR) for any hyponatremia				
Antidepressants (overall)	7	3.160 (1.911–5.225)	<0.001	99.52
SSRIs	6	3.199 (1.750–5.845)	<0.001	99.53
SNRIs	5	3.131 (1.795–5.462)	<0.001	97.71
TCAs	6	1.803 (1.252–2.598)^a^	0.002	91.82
MAOIs	3	3.789 (1.602–8.966)^a^	0.002	50.78
Atypical antidepressants (mirtazapine only)	4	2.818 (1.886–4.211)^a^	<0.001	95.23
Adjusted risk (aOR) for any hyponatremia				
Antidepressants (overall)	6	1.890 (1.573–2.270)	<0.001	91.15
SSRIs	4	2.243 (1.363–3.694)	<0.001	92.94
SNRIs	3	2.009 (1.853–2.179)	<0.001	29.83
TCAs	5	1.753 (1.272–2.416)	<0.001	84.05
Atypical antidepressants (mirtazapine)	3	1.886 (0.782–4.548)	0.158	93.00
Incidence (event rate) for clinically relevant hyponatremia				
Antidepressants (overall)	16	0.0110 (0.0028–0.0421)	<0.001	99.91
SSRIs	13	0.0126 (0.0046–0.0341)	<0.001	99.70
SNRIs	7	0.0257 (0.0055–0.1109)	<0.001	99.52
TCAs	6	0.0093 (0.0013–0.0656)	<0.001	98.79
Atypical antidepressants (mirtazapine only)	5	0.0033 (0.0004–0.0283)	<0.001	98.27
Risk (OR) for clinically relevant hyponatremia				
Antidepressants (overall)	6	2.930 (1.715–5.008)	<0.001	99.60
SSRIs	5	3.085 (1.608–5.920)	<0.001	99.63
SNRIs	4	2.508 (1.343–4.683)	0.003	98.82
TCAs	6	1.803 (1.252–2.598)^a^	0.002	91.82
MAOIs	3	3.789 (1.602–8.966)^a^	0.002	50.78
Atypical antidepressants (mirtazapine only)	4	2.818 (1.886–4.211)^a^	<0.001	95.23

*Note*: Any hyponatremia was defined as a serum sodium <135 mmol/L and clinically relevant hyponatremia as a serum sodium <130 mmol/L or a clinical diagnosis of hyponatremia. For any hyponatremia all studies with an outcome (<135 mmol/L if available, <130 mmol/L if no other outcome was available) were used. The incidences (event rate) and risks (OR) for antidepressant classes were calculated from the pooled outcome measures for the respective antidepressant classes and the outcome measures for specific antidepressants belonging to a certain class.

a For TCAs, MAOIs, and mirtazapine, there were only OR outcomes for clinically relevant hyponatremia.

Meta-regression indicated that 54% of the total between-study variance for event rates was related to the degree of hypoNa (any hyponatremia versus clinically relevant hypoNa, t = −5.91, p < 0.001) and the age group of study population (general adult versus geriatric population, t = 2.58, p = 0.014) (see Supplementary material S1). The available data was insufficient to explore the effects of mean age and gender distribution in meta-regression.

### HypoNa event rate and ORs per antidepressant class and compound

Between antidepressant classes and compounds, we found the event rate of hypoNa to be highest for SNRIs at 7.44% and SSRI at 5.59%, followed by 2.66% for TCAs. The event rate was lowest for atypical antidepressants mirtazapine (1.02%) and trazodone (0.89%) (see Supplementary Figures S7–S10 and S16).

**Figure 2.**
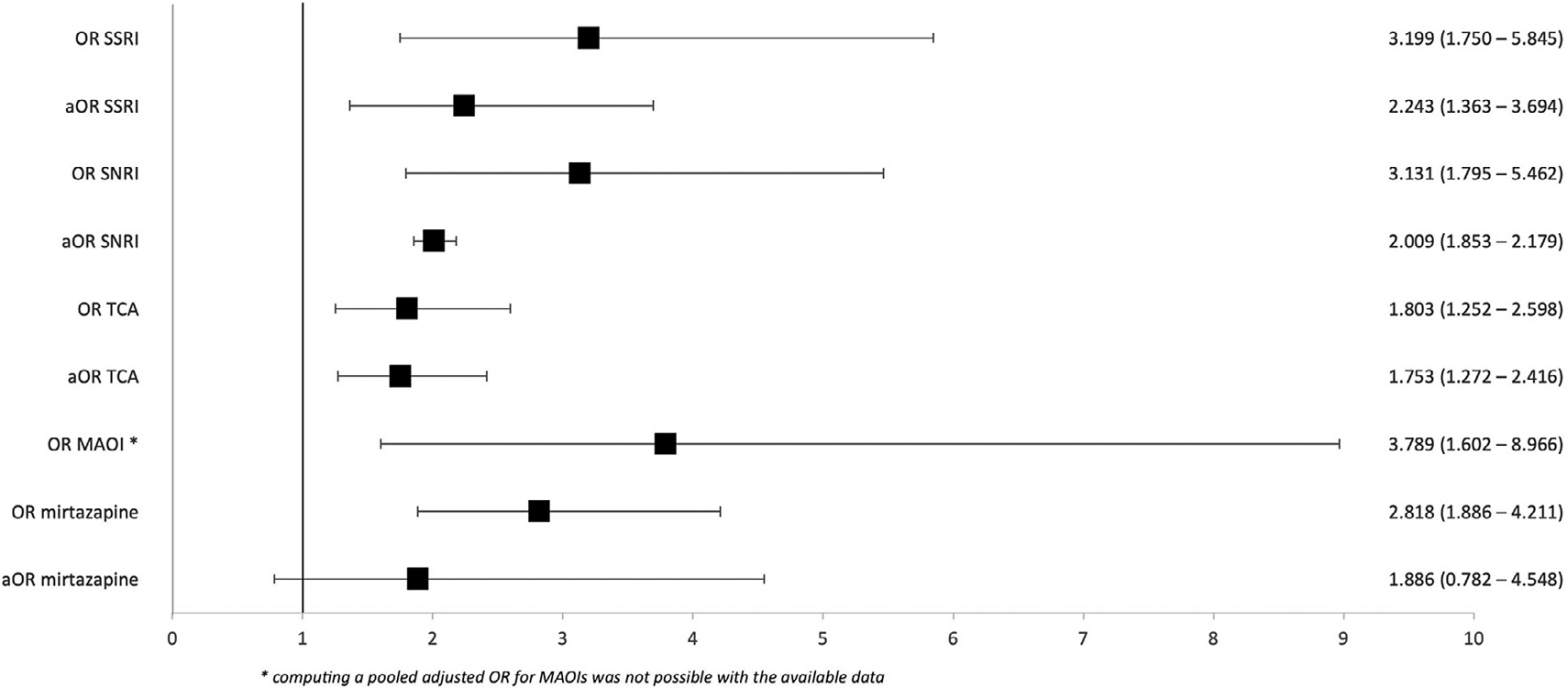
Forest plot summarizing ORs and aORs for any hyponatremia (<135 mmol/L) during treatment with SNRIs, SSRIs, TCAs, MAOIs, and mirtazapine compared to no antidepressant.

We found statistically significantly increased crude and adjusted ORs for SSRIs, SNRIs, and TCAs, and significantly increased crude ORs for MAOIs and mirtazapine. Adjusted ORs were unavailable for MAOIs and non-significant for mirtazapine. The pooled event rates and ORs for every antidepressant class and antidepressant compound for which meta-analysis was possible (≥2 studies) are presented in Tables 2 and 3, respectively. A forest plot of available crude and adjusted ORs is given in Figure 2.

**Table 3.** Pooled incidences (event rates) and odds ratios (OR) for antidepressant compounds

Antidepressant	Number of studies	Event rate or OR (95% confidence interval)	*p*-value	I^2^ (%)
Incidence (event rate) for any hyponatremia				
SSRIs				
Fluoxetine	12	0.0333 (0.0109–0.0971)	<0.001	93.73
Paroxetine	11	0.0542 (0.0138–0.1898)	<0.001	98.74
Sertraline	7	0.0459 (0.0063–0.2681)	0.003	99.21
Escitalopram	6	0.0321 (0.0033–0.2486)	0.004	99.35
Citalopram	3	0.0095 (0.0004–0.2053)	0.006	99.95
Fluvoxamine	4	0.0498 (0.0379–0.0652)	<0.001	00.00
SNRIs				
Venlafaxine	8	0.0699 (0.0105–0.3461)	0.020	98.79
Duloxetine	3	0.0386 (0.0007–0.7104)	0.125	99.44
Milnacipran	3	0.0345 (0.0020–0.3846)	0.022	92.96
TCAs				
Amitriptyline	3	0.0205 (0.0001–0.8430)	0.172	97.88
Atypical antidepressants				
Mirtazapine	8	0.0102 (0.0025–0.0402)	<0.001	97.78
Trazodone	2	0.0089 (0.0000–0.8611)	0.158	99.20
Risk (OR) for any hyponatremia				
SSRIs				
Fluoxetine	4	5.008 (1.713–14.644)	0.006	97.99
Paroxetine	3	4.489 (1.604–12.564)	0.014	98.95
Sertraline	4	3.265 (2.148–4.962)	<0.001	95.71
Escitalopram	3	3.511 (1.508–8.173)	0.004	98.97
Citalopram	3	3.598 (1.826–7.091)	<0.001	99.16
Fluvoxamine	3	2.348 (1.546–3.567)	<0.001	00.00
SNRIs				
Venlafaxine	4	3.899 (2.070–7.344)	<0.001	97.46
Duloxetine	2	3.360 (1.364–8.278)	0.008	97.25
TCAs				
Amitriptyline	2	1.589 (0.487–5.189)	0.443	97.46
Clomipramine	2	4.240 (3.126–5.753)	<0.001	42.23
Imipramine	2	2.059 (0.373–11.375)	0.408	77.17
Trimipramine	2	3.408 (1.610–7.213)	0.001	00.00
Doxepin	2	1.094 (0.400–2.997)	0.861	27.86
MAOIs				
Moclobemide	2	6.100 (3.236–11.500)	<0.001	00.00
Atypical antidepressants				
Mirtazapine	4	2.818 (1.886–4.211)^a^	<0.001	95.23

*Note*: All antidepressant compounds for which meta-analyzable data (>1 study) was available are presented. Any hyponatremia was defined as a serum sodium <135 mmol/L. For any hyponatremia all studies with an outcome (<135 mmol/L if available, <130 mmol/L if no other outcome was available) were used.

a For mirtazapine there were only OR outcomes for clinically relevant hyponatremia.

As between-study heterogeneity was high, we conducted meta-regressions and subgroup analyses to explore potential sources of this heterogeneity. Notably, the degree of hyponatremia (any hypoNa versus clinically relevant hypoNa) significantly impacted event rates for SSRI (total between-groups: Q = 22.9, df = 1, p < 0.001; accounting for 15% of between-study variance), SNRI (Q = 4.97, df = 1, p = 0.026), TCA (Q = 5.31, df = 1, p = 0.021), and mirtazapine (Q = 5.50, df = 1, p = 0.019), with lower rates when only clinically relevant hypoNa was considered (see Supplementary Figures S12–S15). The number of case–control studies was too limited to repeat this analysis for these studies. The results of subgroup analyses contrasting general adult and geriatric study populations were not significant (see Supplementary Figures S22–S25). Our subgroup analysis comparing study contexts indicated that ORs reported in studies based on EHR tended to be lower than the ratios documented in pharmacovigilance and single or multicenter cohort studies, but this difference was not statistically significant (data not shown).

### Head-to-head comparisons of hypoNa risk for antidepressant classes

**Figure 3.**
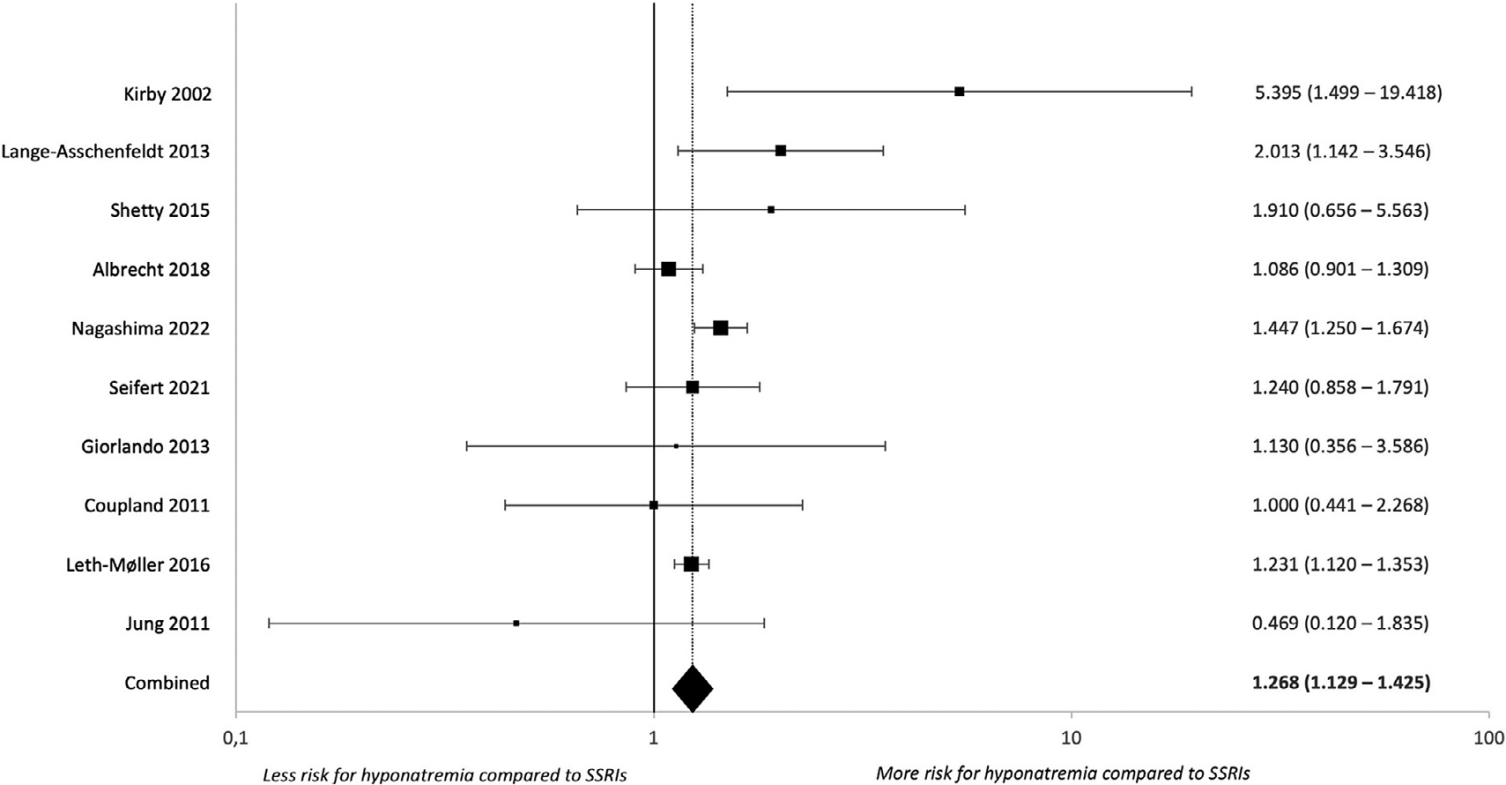
Forest plot for any hyponatremia during treatment with SNRIs compared to SSRIs.

**Figure 4.**
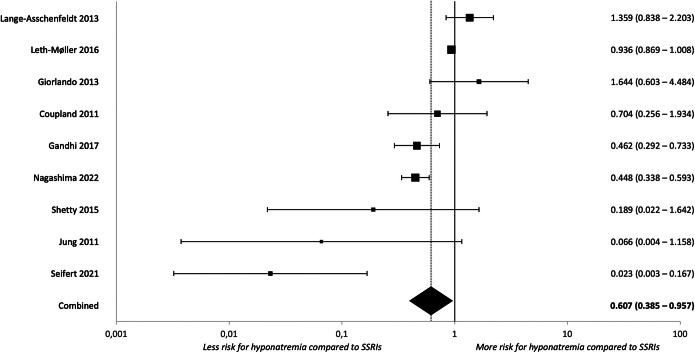
Forest plot for any hyponatremia during treatment with mirtazapine compared to SSRIs.

**Figure 5.**
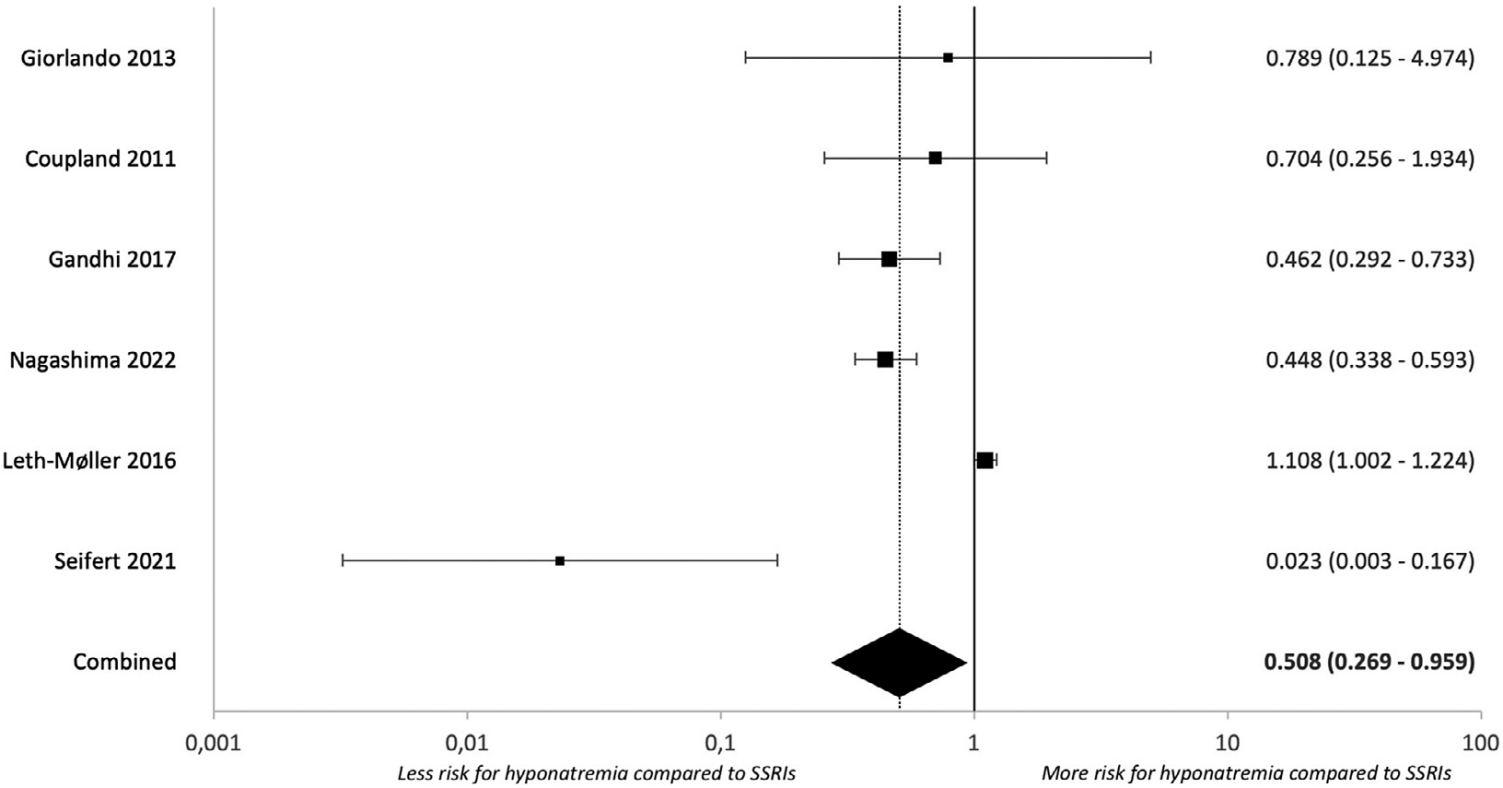
Forest plot for clinically relevant hyponatremia during treatment with mirtazapine compared to SSRIs.

When studies reported outcomes for more than one class of antidepressants, we performed direct head-to-head comparisons to determine the classes’ relative risks of hypoNa as compared to the reference class of SSRI. Results are described in Table 4 and forest plots in Figures 3–5 and Supplementary Figures S44–S47.

**Table 4.** Head-to-head comparison of hyponatremia risk in different antidepressant classes

Compared antidepressant (class)	Number of studies	OR (95% confidence interval)	*p*-value	I^2^ (%)
Risk for any hyponatremia compared to treatment with SSRI				
Mirtazapine*	9	0.607 (0.385–0.957)	0.032	85.53
SNRIs*	10	1.292 (1.120–1.491)	<0.001	45.75
TCAs	9	0.849 (0.624–1.153)	0.294	70.18
Trazodone	3	1.447 (0.156–13.421)	0.745	89.55
Risk for clinically relevant hyponatremia compared to treatment with SSRI				
Mirtazapine*	6	0.508 (0.269–0.959)	0.037	91.65
SNRIs	7	1.154 (0.920–1.447)	0.216	73.59
TCAs	7	0.762 (0.543–1.069)	0.115	72.71
Subgroup analysis for geriatric patients: risk for any hyponatremia compared to treatment with SSRI				
Mirtazapine	4	0.571 (0.373–0.875)	0.010	54.06
SNRIs	5	1.307 (0.985–1.734)	0.064	62.97
TCAs	4	1.028 (0.623–1.696)	0.914	60.74
Subgroup analysis for geriatric patients: risk for clinically relevant hyponatremia compared to treatment with SSRI				
Mirtazapine	4	0.466 (0.370–0.588)	<0.001	00.00
SNRIs	4	1.244 (0.997–1.552)	0.054	50.14
TCAs	3	0.902 (0.587–1.385)	0.637	56.66

*Note*: The statistically significant analyses indicated by a star after the name of the antidepressant compared to SSRIs can be found in the forest plots in Figures 3–5. The forest plots of statistically significant subgroup analyses can be found in the Supplementary material.

We found SNRIs to be significantly more likely to induce hypoNa than SSRIs (k = 10, OR = 1.292 [1.120–1.491], p < 0.001, I^2^ = 45.75%), while mirtazapine was significantly less likely to do so (k = 9, OR = 0.607 [0.385–0.957], p = 0.032, I^2^ = 85.53%). There were no statistically significant differences in the risks associated with TCAs or trazodone versus SSRIs. Our data did not allow to run comparisons with MAOIs and other atypical compounds.

Regarding clinically relevant hypoNa, the risk for mirtazapine was significantly lower compared to SSRIs (k = 6, OR = 0.508 [0.269–0.959], p = 0.037, I^2^ = 91.65%). Results for SNRIs and TCAs were not significantly different from those of SSRIs.

Between-study heterogeneity varied from low to high. Subgroup analyses showed that a higher age of study populations (i.e., geriatric versus non-geriatric participants) and differences in the degree of hyponatremia partly explained the variation. Among geriatric study populations, the findings for mirtazapine remained robust: the agent was still significantly less likely to induce hypoNa (k = 4 studies, OR = 0.571 [0.373–0.875], p = 0.010, I^2^ = 54.06%) or clinically relevant hypoNa (k = 4 studies, OR = 0.466 [0.370–0.588], p < 0.001, I^2^ = 0%) than SSRIs, with moderate to low heterogeneity. We observed a trend of an increased risk for SNRIs compared to SSRIs in geriatric populations, but this association did not reach statistical significance (any hypoNa: k = 5 studies, OR = 1.307 [0.985–1.734], p = 0.064, I^2^ = 62.96; clinically relevant hypoNa: k = 4 studies, OR = 1.244 [0.997–1.552], p = 0.054, I^2^ = 50.14; see Supplementary Figures S46 and S47).

### Bias assessment and meta-regression

Visual inspection of the funnel plots for all event rate studies entered into the primary analysis indicated one potentially missing study left of the mean, but Egger’s test was not significant (t = 1.07, df = 30, p = 0.292; see Supplementary Figures S1–S5). The results of the Newcastle-Ottawa Scale assessment of study quality are reported in Table 1. Meta-regression did not show any effect of study quality on the outcomes. Overall, the results of bias assessment suggest that the studies included in our analyses were not significantly affected by publication bias or study quality.

## Discussion

### Summary of main findings

To the best of our knowledge, the current work represents the first quantitative synthesis of evidence on the risk of adult patients developing hyponatremia when taking antidepressants, comparing both the event rate and ORs for the different classes and compounds of agents reported on. Our findings show that exposure to antidepressants in general is associated with significantly increased odds of both any and clinically relevant hypoNa, even after adjusting for major confounders. No antidepressant class was reliably “safe” in terms of their risk of eliciting hypoNa, with the highest rates for SNRIs and SSRIs, and lower rates for TCAs, mirtazapine, and trazodone. Furthermore, SSRIs, SNRIs, TCAs, MAOIs, and mirtazapine were all statistically significantly associated with increased odds of hypoNa, although these odds were no longer significant for mirtazapine after adjusting for confounders. Unfortunately, for several other atypical and newer antidepressants, the evidence is as yet too sparse to allow valid meta-analytic conclusions to be drawn. In our head-to-head comparisons, we found robust evidence that treatment with mirtazapine carried a lower risk of hypoNa compared to SSRIs, whereas SNRIs were more likely to induce hypoNa than SSRIs. There were no statistically significant differences in the risks associated with TCAs or trazodone versus SSRIs, although in the case of trazodone this was likely related to the low number of available studies. The relative safety of trazodone cannot, therefore, be established yet. The further lack of information on MAOIs or other atypical compounds makes it difficult to determine a comprehensive risk hierarchy at this point.

### Expanding the evidence base

Since our 2014 review, the body of literature on antidepressant-induced hypoNa has grown [3]. Based on this extended body of evidence, and in accordance with our previous findings and the conclusions of several other reports, we consistently found mirtazapine to have a lower risk of hypoNa than SSRIs, confirming that mirtazapine can be a safer therapeutic alternative for hypoNa-prone patients, including those with a geriatric profile [14–16].

Looking for underlying mechanisms of hypoNa, Nagashima et al. examined the correlations between antidepressants and serum sodium levels through their actions on serotonin transporter (SERT) inhibition and found a correlation between decreases in serum sodium levels and the binding affinity for SERT, with mirtazapine showing the lowest binding affinity for SERT [15]. Mirtazapine is known for its atypical receptor profile; more precisely, its primary therapeutic action is presynaptic α2-antagonism; it also blocks several postsynaptic serotonin (5HT) receptors (5HT2A, 5HT2C, 5HT3) and histamine 1 receptors [17].

Although the point values of the event rate and ORs for TCAs were lower than they were for SSRIs and SNRIs, in the head-to-head comparisons with SSRIs, we found no statistically significant differences in the risks associated with TCAs. These discrepancies can be explained by the higher impact of differences in study designs with indirect versus direct (head-to-head) comparisons. Similarly, although the event rates and ORs for SNRIs were similar to those of SSRIs, our head-to-head comparisons uncovered new evidence showing that the risk with SNRIs is higher compared with SSRIs. Previously, several other reviews concluded that the risk was comparable for SNRIs and SSRIs [1, 4, 5]. According to Roxanas et al., venlafaxine may induce hypoNa sooner (mean duration 4 days, within 2–21 days) than an SSRI [18]. Studies that reported on the mean time of hypoNa manifesting in SSRIs mentioned durations of 9 days (within 1–14 days) to 24 days (within 7–42 days) [19, 20]. As to the possible underlying psychopharmacological mechanisms, duloxetine has been demonstrated to have a very high affinity for SERT inhibition [15, 21]. Venlafaxine has less affinity for SERT inhibition than duloxetine and probably has about the same potency for SERT inhibition as paroxetine [22].

In our previous review, we were unable to draw conclusions as to the risks associated with duloxetine, reboxetine, bupropion, and MAOIs [3]. In this meta-analysis we were able to establish that duloxetine and MAOIs are statistically significantly associated with hypoNa (see Tables 2 and 3), but we were still unable to run head-to-head comparisons for these compounds. Despite its interesting non-serotonergic pharmacodynamic profile, the hypoNa risk of bupropion has ever been investigated in only one study, where it did not exceed the risk of no treatment [23]. This seems to support the conclusion Viramontes et al. drew in their review, that bupropion may be an appropriate option for older adults at risk of antidepressant-induced hypoNa [5]. There were no studies reporting outcomes for reboxetine.

### Strengths and limitations of the study

This meta-analysis provides several new, clinically relevant insights into the relative risks of hyponatremia for specific antidepressant compounds and classes, delivering quantitative estimates of event rate and ORs based on the evidence available to date. Nevertheless, several limitations should be considered when interpreting the results, many of them inherent to meta-analytic methods.

Heterogeneity in study design complicated the harmonization and interpretation of results. Both the hypoNa cut-off thresholds and the actual clinical outcomes of low sodium serum levels differed notably among studies (see Table 1). Also, the studies differed considerably with respect to demographic (age, sex), contextual (e.g., main diagnosis and treatment setting), and methodological aspects.

Most of the study participants were of older age and/or receiving treatment in a general hospital, which limits the extent to which the findings can be applied to younger populations without somatic comorbidity. Furthermore, our dataset included several pharmacovigilance studies (see Table 1). Besides the higher risk of reporting bias in pharmacovigilance studies, the pooling of reporting odds ratios (ROR) from case-non-case analyses in two studies [22, 31] with OR from regular case–control analyses also presented a methodological challenge. While our subgroup analyses for the types of study contexts (pharmacovigilance studies versus EHR versus cohort/case–control studies) were unfortunately lacking in power, all of our main findings remained robust in sensitivity analyses after exclusion of pharmacovigilance studies (see Supplementary Table S7).

Although we were able to explain significant proportions of the overall heterogeneity in subgroup analyses and meta-regressions of hypoNa cut-off and geriatric patient profiles, other factors that we were unable to examine will undoubtedly have contributed to the variability in the results.

Generalizability of results is also impacted because of limitations in the original studies. One notable example is that several cohort studies [18, 24–30] and case–control studies [10, 23, 31–33] did not specify whether their patients were exposed to a single or multiple antidepressants during the study period. Our calculated effect sizes, therefore, assumed that patients only took one antidepressant during the study period, which could have translated into inflated event rates or ORs for antidepressants such as mianserin, mirtazapine, bupropion, or trazodone that are often used as add-on therapies in clinical practice. Our result for pooled ORs for MAOIs is mostly based on studies reporting on moclobemide and, to a lesser extent, iproniazid. The given pooled ORs for MAOIs are therefore not representative of the risk of HypoNa with irreversible MAOIs.

Furthermore, case–control studies did not always differentiate between newly started or ongoing treatments; retrospective cohort studies did not necessarily apply routine laboratory testing; and prospective cohort studies had varying follow-up periods (ranging from 4 up to 26 weeks). A final limitation follows out of the demonstration by Leth-Møller et al. that the likelihood of a blood sample being taken from patients treated with a specific antidepressant compound contributes to the reported incidences within retrospective studies [13].

### Clinical relevance and future research

Evidence on the differences in the risks of hypoNa provided in this work can directly support the prescription and monitoring practices of mental health practitioners when it comes to antidepressants, especially when it concerns older or vulnerable patients for whom the early recognition of drug-induced hypoNa can be of vital importance. One should, however, remember hypoNa is not the only adverse effect of antidepressants. In a recent expert guideline, monitoring the electrolyte balance is recommended as best practice after initiation of a new pharmacological agent, including but not limited to antidepressants, with a known risk of increasing hypoNa in order to prevent serious complications [16]. Monitoring serum sodium for 2–4 weeks after the start of antidepressant treatment in hypoNa-prone patients is appropriate for every antidepressant but especially so for SNRIs and SSRIs.

We need hypoNa-focused research that (also) includes antidepressants for which there is as yet limited evidence, especially bupropion, which in one study stood out because of its safety in this respect. Larger studies comparing the risk profiles of multiple antidepressant compounds and classes are required to replicate the current findings and expand the evidence base, particularly regarding the novel finding of a higher risk from SNRIs compared to SSRIs. Finally, determining events based on routine laboratory monitoring rather than relying solely on record linkages will provide less biased estimates of the risks of hypoNa for particular antidepressants and thus help inform clinical decision-making.

## Conclusion

Although between-study heterogeneity was high, this review and meta-analysis recorded the highest event rates of (any) hyponatremia for SNRIs and SSRIs, followed by TCAs, and the lowest event rates for mirtazapine and trazodone. The highest ORs of any hypoNa were found for MAOIs, followed by SNRIs and SSRIs, and the lowest for mirtazapine and TCAs. Head-to-head comparisons showed that SNRIs are more and mirtazapine less likely to induce hypoNa, compared to SSRIs. Thus, when weighing the risk of hypoNa as such, mirtazapine should be considered as the treatment of choice, and SNRIs should be prescribed more cautiously than SSRIs and TCAs, especially in elderly patients.

## Supporting information

Gheysens et al. supplementary material Gheysens et al. supplementary material

## Supplementary material

The supplementary material for this article can be found at https://doi.org/10.1192/j.eurpsy.2024.11.
