# Supplementary material for: The risk of antidepressant-induced hyponatremia: A meta-analysis of antidepressant classes and compounds
Source: Eur Psychiatry. 2024 Feb 26;67(1):e20. doi: 10.1192/j.eurpsy.2024.11 (PMC10966618; doi:10.1192/j.eurpsy.2024.11)
Supplement: Gheysens et al. supplementary material [file S0924933824000117sup001.docx]

Appendix

The risk of antidepressant-induced hyponatremia: A meta-analysis of antidepressant classes and compounds

[Supplementary Table 1. PRISMA checklist 4](#_Toc155632398)

[Supplementary Table 2. Search strings on PubMed and Web of Science 7](#_Toc155632399)

[Supplementary Table 3. ATC-codes 8](#_Toc155632400)

[Supplementary Table 4. List of extracted variables 9](#_Toc155632401)

[Supplementary Table 5. Comments on inclusion process 10](#_Toc155632402)

[Supplementary Table 6. Overview databases per eligible study 13](#_Toc155632403)

[Supplementary Table 7: Sensitivity analyses with exclusion of pharmacovigilance studies (primary analyses) 15](#_Toc155632404)

[Supplementary Figure 1. Funnel plot and Egger test event rate (any hyponatremia) 16](#_Toc155632405)

[Supplementary Figure 2. Funnel plot odds ratio (any hyponatremia) 17](#_Toc155632406)

[Supplementary Figure 3. Funnel plot head-to-head comparison mirtazapine-SSRI (any hyponatremia) 18](#_Toc155632407)

[Supplementary Figure 4. Funnel plot head-to-head comparison SNRI-SSRI (any hyponatremia) 19](#_Toc155632408)

[Supplementary Figure 5. Funnel plot head-to-head comparison TCA-SSRI (any hyponatremia) 20](#_Toc155632409)

[Supplementary Figure 6. Forest plot event rates overall antidepressants (any hyponatremia) 21](#_Toc155632410)

[Supplementary Figure 7. Forest plot event rates SSRI (any hyponatremia) 22](#_Toc155632411)

[Supplementary Figure 8. Forest plot event rates SNRI (any hyponatremia) 23](#_Toc155632412)

[Supplementary Figure 9. Forest plot event rates TCA (any hyponatremia) 24](#_Toc155632413)

[Supplementary Figure 10. Forest plot event rates mirtazapine (any hyponatremia) 25](#_Toc155632414)

[Supplementary Figure 11. Forest plot event rates overall antidepressants (clinically relevant hyponatremia) 26](#_Toc155632415)

[Supplementary Figure 12. Forest plot event rates SSRIs (clinically relevant hyponatremia) 27](#_Toc155632416)

[Supplementary Figure 13. Forest plot event rates SNRIs (clinically relevant hyponatremia) 28](#_Toc155632417)

[Supplementary Figure 14. Forest plot event rates TCAs (clinically relevant hyponatremia) 29](#_Toc155632418)

[Supplementary Figure 15. Forest plot event rates mirtazapine (clinically relevant hyponatremia) 30](#_Toc155632419)

[Supplementary Figure 16. Forest plot event rates antidepressant compounds (any hyponatremia) 31](#_Toc155632420)

[Supplementary Figure 17. Forest plot event rates overall antidepressants for subgroups by cut-off 32](#_Toc155632421)

[Supplementary Figure 18. Forest plot event rates SSRIs for subgroups by cut-off 33](#_Toc155632422)

[Supplementary Figure 19. Forest plot event rates SNRIs for subgroups by cut-off 34](#_Toc155632423)

[Supplementary Figure 20. Forest plot event rates TCAs for subgroups by cut-off 35](#_Toc155632424)

[Supplementary Figure 21. Forest plot event rates mirtazapine for subgroups by cut-off 36](#_Toc155632425)

[Supplementary Figure 22. Forest plot event rates overall antidepressants (any hyponatremia) for subgroups by age 37](#_Toc155632426)

[Supplementary Figure 23. Forest plot event rates SSRIs (any hyponatremia) for subgroups by age 38](#_Toc155632427)

[Supplementary Figure 24. Forest plot event rates overall antidepressants (clinically relevant hyponatremia) for subgroups by age 39](#_Toc155632428)

[Supplementary Figure 25. Forest plot event rates SNRIs (clinically relevant hyponatremia) for subgroups by age 40](#_Toc155632429)

[Supplementary Figure 26. Forest plot crude odds ratios overall antidepressants (any hyponatremia) 41](#_Toc155632430)

[Supplementary Figure 27. Forest plot crude odds ratios SSRIs (any hyponatremia) 42](#_Toc155632431)

[Supplementary Figure 28. Forest plot crude odds ratios SNRIs (any hyponatremia) 43](#_Toc155632432)

[Supplementary Figure 29. Forest plot crude odds ratios TCAs (any hyponatremia)* 44](#_Toc155632433)

[Supplementary Figure 30. Forest plot crude odds ratios MAOIs (any hyponatremia)* 45](#_Toc155632434)

[Supplementary Figure 31. Forest plot crude odds ratios mirtazapine (any hyponatremia)* 46](#_Toc155632435)

[Supplementary Figure 32. Forest plot crude odds ratios overall antidepressants (clinically relevant hyponatremia) 47](#_Toc155632436)

[Supplementary Figure 33. Forest plot crude odds ratios SSRI (clinically relevant hyponatremia) 48](#_Toc155632437)

[Supplementary Figure 34. Forest plot odds ratios crude SNRI (clinically relevant hyponatremia) 49](#_Toc155632438)

[Supplementary Figure 35. Forest plot odds ratios antidepressant compounds (any hyponatremia) 50](#_Toc155632439)

[Supplementary Figure 36. Forest plot adjusted odds ratios overall antidepressants (any hyponatremia) 51](#_Toc155632440)

[Supplementary Figure 37. Forest plot adjusted odds ratios SSRIs (any hyponatremia) 52](#_Toc155632441)

[Supplementary Figure 38. Forest plot adjusted odds ratios SNRIs (any hyponatremia) 53](#_Toc155632442)

[Supplementary Figure 39. Forest plot adjusted odds ratios TCAs (any hyponatremia) 54](#_Toc155632443)

[Supplementary Figure 40. Forest plot adjusted odds ratios mirtazapine (any hyponatremia) 55](#_Toc155632444)

[Supplementary Figure 41. Forest plot odds ratios overall antidepressants (any hyponatremia) for subgroups by age 56](#_Toc155632445)

[Supplementary Figure 42. Forest plot adjusted odds ratios any antidepressant (any hyponatremia) for subgroups by age 57](#_Toc155632446)

[Supplementary Figure 43. Forest plot adjusted odds ratios any antidepressant (clinically relevant hyponatremia) 58](#_Toc155632447)

[Supplementary Figure 44. Forest plot head-to-head comparison (any hyponatremia) 59](#_Toc155632448)

[Supplementary Figure 45. Forest plot head-to-head comparison (clinically relevant hyponatremia) 60](#_Toc155632449)

[Supplementary Figure 46. Forest plot head-to-head comparison (any hyponatremia, only geriatric subgroup) 61](#_Toc155632450)

[Supplementary Figure 47. Forest plot head-to-head comparison (clinically relevant hyponatremia, only geriatric subgroup) 62](#_Toc155632451)

[Supplementary Material 1. Meta-regression event rates overall antidepressants (any hyponatremia) 63](#_Toc155632452)

[Supplementary Material 2: updates to PROSPERO protocol 64](#_Toc155632453)

# **Supplementary Table 1.** PRISMA checklist

| **Section and Topic** | **Item #** | **Checklist item** | **Location where item is reported** |
| --- | --- | --- | --- |
| **TITLE** | | |  |
| Title | 1 | Identify the report as a systematic review. | Title |
| **ABSTRACT** | | |  |
| Abstract | 2 | See the PRISMA 2020 for Abstracts checklist. | Abstract |
| **INTRODUCTION** | | |  |
| Rationale | 3 | Describe the rationale for the review in the context of existing knowledge. | 1. Introduction, paragraphs 1-3 |
| Objectives | 4 | Provide an explicit statement of the objective(s) or question(s) the review addresses. | 1. Introduction, paragraph 4 |
| **METHODS** | | |  |
| Eligibility criteria | 5 | Specify the inclusion and exclusion criteria for the review and how studies were grouped for the syntheses. | 2.2 Methods, Eligibility criteria |
| Information sources | 6 | Specify all databases, registers, websites, organisations, reference lists and other sources searched or consulted to identify studies. Specify the date when each source was last searched or consulted. | 2.1 Methods Search strategy and Figure 1. PRISMA flow chart |
| Search strategy | 7 | Present the full search strategies for all databases, registers and websites, including any filters and limits used. | Supplementary Table 2. |
| Selection process | 8 | Specify the methods used to decide whether a study met the inclusion criteria of the review, including how many reviewers screened each record and each report retrieved, whether they worked independently, and if applicable, details of automation tools used in the process. | 2.1 Methods, Search strategy, last paragraph and Figure 1. PRISMA flow chart |
| Data collection process | 9 | Specify the methods used to collect data from reports, including how many reviewers collected data from each report, whether they worked independently, any processes for obtaining or confirming data from study investigators, and if applicable, details of automation tools used in the process. | 2.4 Methods, Data extraction and processing |
| Data items | 10a | List and define all outcomes for which data were sought. Specify whether all results that were compatible with each outcome domain in each study were sought (e.g. for all measures, time points, analyses), and if not, the methods used to decide which results to collect. | 2.3 Methods, Outcome Measures and Supplementary Table 3. |
|  | 10b | List and define all other variables for which data were sought (e.g. participant and intervention characteristics, funding sources). Describe any assumptions made about any missing or unclear information. | 2.4 Methods, Data extraction and processing and Supplementary Table 3. |
| Study risk of bias assessment | 11 | Specify the methods used to assess risk of bias in the included studies, including details of the tool(s) used, how many reviewers assessed each study and whether they worked independently, and if applicable, details of automation tools used in the process. | 2.5 Study quality |
| Effect measures | 12 | Specify for each outcome the effect measure(s) (e.g. risk ratio, mean difference) used in the synthesis or presentation of results. | 2.3 Methods, Outcome measures and Supplementary Table 3. |
| Synthesis methods | 13a | Describe the processes used to decide which studies were eligible for each synthesis (e.g. tabulating the study intervention characteristics and comparing against the planned groups for each synthesis (item #5)). | 2.6 Methods, Data synthesis and analysis |
|  | 13b | Describe any methods required to prepare the data for presentation or synthesis, such as handling of missing summary statistics, or data conversions. | 2.4 Methods, Data extraction and processing, second paragraph |
|  | 13c | Describe any methods used to tabulate or visually display results of individual studies and syntheses. | 2.6 Methods, Data synthesis and analysis, first paragraph |
|  | 13d | Describe any methods used to synthesize results and provide a rationale for the choice(s). If meta-analysis was performed, describe the model(s), method(s) to identify the presence and extent of statistical heterogeneity, and software package(s) used. | 2.6 Methods, Data synthesis and analysis |
|  | 13e | Describe any methods used to explore possible causes of heterogeneity among study results (e.g. subgroup analysis, meta-regression). | 2.6 Methods, Data synthesis and analysis |
|  | 13f | Describe any sensitivity analyses conducted to assess robustness of the synthesized results. | 2.6 Methods, Data synthesis and analysis |
| Reporting bias assessment | 14 | Describe any methods used to assess risk of bias due to missing results in a synthesis (arising from reporting biases). | 2.6 Methods, Data synthesis and analysis |
| Certainty assessment | 15 | Describe any methods used to assess certainty (or confidence) in the body of evidence for an outcome. | 2.6 Methods, Data synthesis and analysis |
| **RESULTS** | | |  |
| Study selection | 16a | Describe the results of the search and selection process, from the number of records identified in the search to the number of studies included in the review, ideally using a flow diagram. | 3.1. Results, Study selection, first paragraph, and Figure 1. PRISMA flow chart |
|  | 16b | Cite studies that might appear to meet the inclusion criteria, but which were excluded, and explain why they were excluded. | Supplementary Table 4. |
| Study characteristics | 17 | Cite each included study and present its characteristics. | Table 1. |
| Risk of bias in studies | 18 | Present assessments of risk of bias for each included study. | Table 1. |
| Results of individual studies | 19 | For all outcomes, present, for each study: (a) summary statistics for each group (where appropriate) and (b) an effect estimate and its precision (e.g. confidence/credible interval), ideally using structured tables or plots. | Results, Table 2, 3 and 4, Supplementary Figure 6-16 |
| Results of syntheses | 20a | For each synthesis, briefly summarise the characteristics and risk of bias among contributing studies. | Results, Table 2, 3 and 4, Supplementary Figure 1-5 |
|  | 20b | Present results of all statistical syntheses conducted. If meta-analysis was done, present for each the summary estimate and its precision (e.g. confidence/credible interval) and measures of statistical heterogeneity. If comparing groups, describe the direction of the effect. | Results, Table 2, 3 and 4, Figure 2, 3 and 4 |
|  | 20c | Present results of all investigations of possible causes of heterogeneity among study results. | Results, Supplementary Figures 1-5 |
|  | 20d | Present results of all sensitivity analyses conducted to assess the robustness of the synthesized results. | Results, Table 2, 3 and 4 |
| Reporting biases | 21 | Present assessments of risk of bias due to missing results (arising from reporting biases) for each synthesis assessed. | Results, Supplementary Figure 1-5 |
| Certainty of evidence | 22 | Present assessments of certainty (or confidence) in the body of evidence for each outcome assessed. | Results, Table 2, 3 and 4 |
| **DISCUSSION** | | |  |
| Discussion | 23a | Provide a general interpretation of the results in the context of other evidence. | 4. Discussion, paragraph 1 - 3 |
|  | 23b | Discuss any limitations of the evidence included in the review. | 4. Discussion, paragraph 4 - 5 |
|  | 23c | Discuss any limitations of the review processes used. | 4. Discussion, paragraph 4 – 5 |
|  | 23d | Discuss implications of the results for practice, policy, and future research. | 4. Discussion, paragraph 6 - 7 |
| **OTHER INFORMATION** | | |  |
| Registration and protocol | 24a | Provide registration information for the review, including register name and registration number, or state that the review was not registered. | 2. Methods |
|  | 24b | Indicate where the review protocol can be accessed, or state that a protocol was not prepared. | Protocol as supplementary document |
|  | 24c | Describe and explain any amendments to information provided at registration or in the protocol. | Updates to protocol as supplementary document |
| Support | 25 | Describe sources of financial or non-financial support for the review, and the role of the funders or sponsors in the review. | 7. Financial support |
| Competing interests | 26 | Declare any competing interests of review authors. | 8. Conflicts of interest |
| Availability of data, code and other materials | 27 | Report which of the following are publicly available and where they can be found: template data collection forms; data extracted from included studies; data used for all analyses; analytic code; any other materials used in the review. | Collected data is not publicly available |

# **Supplementary Table 2.** Search strings on PubMed and Web of Science

| Platform | search strategy |
| --- | --- |
| PubMed | (((Hyponatremia[MeSH Terms]) OR (Inappropriate ADH Syndrome[MeSH Terms])) AND (Antidepressive agents[MeSH Terms])) |
| Web of Science | (TS=((antidepress* OR SSRI OR reuptake inhibitor OR SNRI OR TCA OR tricyclic OR MAO OR mono-amine oxidase OR reboxetine* OR mirtazapin* OR trazodon* OR mianserin* OR agomelatin* OR bupropion OR vortioxetin* OR *venlafaxin* OR duloxetin* OR *ketamin*) AND (hyponatremia OR hyponatraemia OR SIADH OR inappropriate ADH))) |

# **Supplementary Table 3. ATC-codes**

| **SSRIs** | **N06AB** |
| --- | --- |
| fluoxetine | N06AB03 |
| citalopram | N06AB04 |
| paroxetine | N06AB05 |
| sertraline | N06AB06 |
| fluvoxamine | N06AB08 |
| escitalopram | N06AB10 |
| **SNRIs** | **N06AX** |
| venlafaxine | N06AX16 |
| milnacipran | N06AX17 |
| duloxetine | N06AX21 |
| desvenlafaxine | N06AX23 |
| levomilnacipran | N06AX28 |
| **TCAs** | **N06AA** |
| imipramine | N06AA02 |
| clomipramine | N06AA04 |
| trimipramine | N06AA06 |
| amitriptyline | N06AA09 |
| nortriptyline | N06AA10 |
| doxepin | N06AA12 |
| maprotiline | N06AA21 |
| **MAOIs** | **N06AF & N06AG** |
| iproniazid | N06AF05 |
| moclobemide | N06AG02 |
| **atypical antidepressants** | **N06AX** |
| trazodone | N06AX05 |
| mirtazapine | N06AX11 |
| bupropion | N06AX12 |
| reboxetine | N06AX18 |
| agomelatine | N06AX22 |
| vortioxetine | N06AX26 |

| **Supplementary Table 4.** List of extracted variables |
| --- |
| study design (case-control, retrospective or prospective cohort, trial, cross-sectional) |
| source of information |
| country |
| raw data (e.g. frequency of exposure to antidepressant, divided by name and class, in cases and controls in the case control studies and the frequency data of hyponatremia events in exposed and nonexposed individuals in the cohort studies) |
| crude and adjusted OR, RR or HR and their 95%CI |
| covariates used in multivariate analyses (e.g., comedication, comorbidities, ..) |
| mean dose of antidepressant used |
| mean duration of antidepressant use |
| indication for the antidepressant |
| cut off value for hyponatremia (mmol/L) |
| clinical outcome of hyponatremia (asymptomatic, symptomatic, hospitalization, mortality) |
| routine lab monitoring or not |
| follow up period |
| clinical context of patients |
| nature of the control group |
| mean age |
| percentages of female study participants |

| **Supplementary Table 5.** Comments on inclusion process | |
| --- | --- |
| Kirby et al.[1] | In the qualitative analysis in the paper by De Picker et al.[2] this paper was classified as a retrospective cohort study. The data that was extracted (OR, e.g.) out of the study for this meta-analysis were however outcomes of a study type better classified as a case control study. |
| Leth-Møller et al.[3] | This study was included both as a retrospective cohort study as a case control study.  We have used the event rate for overall antidepressants reported in this study for all possible analyses. We could however only use the event rates for specific antidepressant compounds for the head-to-head analyses since comparisons in these analyses are done within the same study. We could not compare the event rates with event rates in other studies because unlike other studies the n are the number of events and N are the number of blood analyses in the study by Leth-Møller et al., and not necessarily the number of exposed participants. |
| Rochoy et al.[4] Revol et al.[5] Montastruc et al.[6] | These three pharmacovigilance studies had an overlap between their databases (see Supplementary Table 6). We have chosen to include all three studies because of their complementarity in reported outcomes. Montastruc et al. and Revol et al. reported event rates for the time periods 2008 and 2011-2013, respectively. The study by Rochoy et al. reported odds ratios for the time period 2004-2013.  *For the study by Revol et al. we have included the data in Table 3 in the paper. |
| Farmand et al.[7], Mannheimer et al.[8] | These studies were seen as one study since they were based on the exact same database and largely reported the same outcome measures. Encountering small differences between ORs for SSRIs in these two studies, we have opted to use the values given in the study by Farmand et al.[7].  We based our values on the number of cases and controls and the ORs given in the corrigendum[9] and the number of exposed cases and controles given in the original paper by Farmand et al.[7]. Since Farmand et al. updated the adjusted ORs in their corrigendum for the two separate groups (newly initiated antidepressants and ongoing antidepressants) albeit not for the total number of cases and controls per antidepressant, we decided to not include the faulty adjusted ORs from this study. |
| Wilkinson et al.[10] | This study was not included as a case control study since it did not deliver odds ratios for our research question, albeit we could include it as a retrospective cohort study. |
| Degner et al.[11], Letmaier et al[12]. | These studies were excluded because they were based on overlapping databases with the more recent and complete publication by Seifert et al.[13]. |
| Movig et al.[14] | This study was excluded because it compared the occurrence of hyponatremia during treatment with SSRIs to treatment with other antidepressants and not to no exposure to antidepressants at all. |
| Takeda et al.[15] | This study was excluded because it reported odds ratios for clinicians reporting hyponatremia specifically due to therapy with an antidepressant within a pharmacovigilance program which is not comparable to (reporting) odds ratios for hyponatremia during treatment with an antidepressant. |
| Nagashima et al.[16] | In this study we could only extract enough data to include a certain antidepressant compound for the antidepressants mentioned in Figure 4.A and 4.B in the study paper. |
| Noohi et al.[17] | For this study it was also possible to extract data for clinically relevant hypoNa (serum sodium < 130mmol/L) for both an SSRI- and a non-SSRI-group, incidences of 1.9% and 2.6% respectively. We have chosen to not include this data since the calculated number of events with these incidences is less than zero. |
| Coupland et al.[18] | In their longitudinal follow-up study, Coupland and colleagues provided several metrics which allowed us to generate 1-year adjusted event rate estimates for the meta-analysis, although these data represent approximations rather than exact incidences, as they assume that the distribution of events remained uniform over the 5-year follow-up period. We opted to calculate 1-year rather than 5-year follow-up outcomes, as these would increase comparability with other studies in the meta-analysis. To estimate of the sample size in each antidepressant category over a one-year follow-up window, we used data provided in the full report of the study.  We estimated the average number of events in one year of follow-up time by dividing the number of events recorded over the total follow-up period by the average follow-up time (5±3.3 years, Table 15). We then divided the average number of events for a one-year follow-up period by the absolute risk (%) of hyponatremia at 1 year from the baseline date per class and AD, reported in Table 108 in the original paper. These risks were adjusted for confounders (Adjusted for gender, age (5-year bands), year, depression severity, depression before age 65 years, smoking status, Townsend deprivation score, CHD, diabetes, hypertension, stroke, cancer, dementia, epilepsy/seizures, Parkinson’s disease, hypothyroidism, obsessive–compulsive disorder, statins, NSAIDs, antipsychotics, aspirin, antihypertensives, anticonvulsants and hypnotics/anxiolytics.).  - SSRI: (383 events / 5 years) / 0,0044 = 17.409,09 - TCA: (155 events / 5 years) / 0,0030 = 10.333,33 - Other: (62 events / 5 years) ) / 0,0037 = 3351,35 - Unexposed to AD: (503 events / 5 years) / 0,0029 = 34689,66  For mirtazapine and venlafaxine, the number of events was not reported separately but as part of an “other” category (cfr above). We therefore estimated their sample sizes using the relative proportion of both compounds prescriptions’ within the other category:  - mirtazapine 5258/9944 prescriptions = 38.15% of ‘other’ = 1278,41  - venlafaxine 4686/9944 prescriptions = 42.17% of ‘other’ = 1413,12 |
| Mazhar et al.[19] Leth-Møller et al.[3] Takeda et al.[15] | If confidence intervals were too asymmetric (despite a fudge ratio of 2.0 in CMA), we widened it until analysis was possible. We have done this for the aORs for sertraline, paroxetine, SSRI, duloxetine, SNRI, imipramine, trimipramine and doxepin in the study by Mazhar and colleagues for mirtazapine in the study by Leth-Møller and colleagues; for duloxetine in the study by Takeda and colleagues. |
| Shetty et al.[20] | The event rates for antidepressant compounds for which the sample size was below 2 could only be used in the head-to-head analyses (TCA and trazodone). |

References supplementary Table 5.

[1] Kirby D, Harrigan S, Ames D. Hyponatraemia in elderly psychiatric patients treated with Selective Serotonin Reuptake Inhibitors and venlafaxine: a retrospective controlled study in an inpatient unit. Int J Geriatr Psychiatr. 2002;17(3):231-7. <https://doi.org/10.1002/gps.591>.

[2] De Picker L, Van den Eede F, Dumont G, Moorke G, Sabbe BGC. Antidepressants and the Risk of Hyponatremia: A Class-by-Class Review of Literature. Psychosomatics. 2014;55(6):536-47.

[3] Leth-Møller KB, Hansen AH, Torstensson M, Andersen SE, Odum L, Gislasson G, et al. Antidepressants and the risk of hyponatremia: a Danish register-based population study. BMJ Open. 2016;6(5):8:e011200. <https://doi.org/10.1136/bmjopen-2016-011200>.

[4] Rochoy M, Zakhem-Stachera C, Bene J, Berkhout C, Gautier S, Reseau Francais Ctr R. Antidepressive agents and hyponatremia: A literature review and a case/non-case study in the French Pharmacovigilance database. Therapie. 2018;73(5):389-98. <https://doi.org/10.1016/j.therap.2018.02.006>.

[5] Revol R, Rault C, Polard E, Bellet F, Guy C. Hyponatremia associated with SSRI/NRSI: Descriptive and comparative epidemiological study of the incidence rates of the notified cases from the data of the French National Pharmacovigilance Database and the French National Health Insurance. Enceph-Rev Psychiatr Clin Biol Ther. 2018;44(3):291-6. <https://doi.org/10.1016/j.encep.2017.09.003>.

[6] Montastruc F, Sommet A, Bondon-Guitton E, Durrieu G, Bui E, Bagheri H, et al. The importance of drug-drug interactions as a cause of adverse drug reactions: a pharmacovigilance study of serotoninergic reuptake inhibitors in France. Eur J Clin Pharmacol. 2012;68(5):767-75. <https://doi.org/10.1007/s00228-011-1156-7>.

[7] Farmand S, Lindh JD, Calissendorff J, Skov J, Falhammar H, Nathanson D, et al. Differences in Associations of Antidepressants and Hospitalization Due to Hyponatremia. Am J Med. 2018;131(1):56-63. <https://doi.org/10.1016/j.amjmed.2017.07.025>.

[8] Mannheimer B, Falhammar H, Calissendorff J, Skov J, Lindh JD. Time-dependent association between selective serotonin reuptake inhibitors and hospitalization due to hyponatremia. J Psychopharmacol. 2021;35(8):928-33:02698811211001082. <https://doi.org/10.1177/02698811211001082>.

[9] Farmand S, Lindh JD, Calissendorff J, Skov J, Falhammar H, Nathanson D, et al. Corrigendum to "Differences in Associations of Antidepressants and Hospitalization Due to Hyponatremia" AmJMed, 131(01);56-63. Am J Med. 2021;134(4):554. <https://doi.org/10.1016/j.amjmed.2020.12.003>.

[10] Wilkinson TJ, Begg EJ, Winter AC, Sainsbury R. Incidence and risk factors for hyponatraemia following treatment with fluoxetine or paroxetine in elderly people. Br J Clin Pharmacol. 1999;47(2):211-7. <https://doi.org/10.1046/j.1365-2125.1999.00872.x>.

[11] Degner D, Grohmann R, Kropp S, Ruther E, Bender S, Engel RR, et al. Severe adverse drug reactions of antidepressants: Results of the German multicenter drug surveillance program AMSP. Pharmacopsychiatry. 2004;37:S39-S45. <https://doi.org/10.1055/s-2004-815509>.

[12] Letmaier M, Painold A, Holl AK, Vergin H, Engel R, Konstantinidis A, et al. Hyponatraemia during psychopharmacological treatment: results of a drug surveillance programme. Int J Neuropsychopharmacol. 2012;15(6):739-48. <https://doi.org/10.1017/s1461145711001192>.

[13] Seifert J, Letmaier M, Greiner T, Schneider M, Deest M, Eberlein CK, et al. Psychotropic drug-induced hyponatremia: results from a drug surveillance program-an update. J Neural Transm. 2021;128(8):1249-64. <https://doi.org/10.1007/s00702-021-02369-1>.

[14] Movig KL, Leufkens HG, Lenderink AW, van den Akker VG, Hodiamont PP, Goldschmidt HM, et al. Association between antidepressant drug use and hyponatraemia: a case-control study. Br J Clin Pharmacol. 2002;53(4):363-9. <https://doi.org/10.1046/j.1365-2125.2002.01550.x>.

[15] Takeda K, Kobayashi C, Nakai T, Oishi T, Okada A. Analysis of the Frequency and Onset Time of Hyponatremia/Syndrome of Inappropriate Antidiuretic Hormone Induced by Antidepressants or Antipsychotics. Ann Pharmacother. 2021:6:10600280211030270. <https://doi.org/10.1177/10600280211030270>.

[16] Nagashima T, Hayakawa T, Akimoto H, Minagawa K, Takahashi Y, Asai S. Identifying Antidepressants Less Likely to Cause Hyponatremia: Triangulation of Retrospective Cohort, Disproportionality, and Pharmacodynamic Studies. Clinical Pharmacology & Therapeutics. 2022;111(6):1258-67. <https://doi.org/10.1002/cpt.2573>.

[17] Noohi S, Do A, Elie D, Mahdanian AA, Yu C, Segal M, et al. Selective Serotonin Re-Uptake Inhibitors and Hyponatremia in Acutely Medically-Ill Inpatients. Curr Drug Saf. 2016;11(2):121-7. <https://doi.org/10.2174/1574886311666160226131603>.

[18] Coupland C, Dhiman P, Morriss R, Arthur A, Barton G, Hippisley-Cox J. Antidepressant use and risk of adverse outcomes in older people: population based cohort study. BMJ-British Medical Journal. 2011;343:15:d4551. <https://doi.org/10.1136/bmj.d4551>.

[19] Mazhar F, Pozzi M, Gentili M, Scatigna M, Clementi E, Radice S, et al. Association of Hyponatraemia and Antidepressant Drugs: A Pharmacovigilance-Pharmacodynamic Assessment Through an Analysis of the US Food and Drug Administration Adverse Event Reporting System (FAERS) Database. CNS Drugs. 2019;33(6):581-92. <https://doi.org/10.1007/s40263-019-00631-5>.

[20] Shetty H. M. MK, Sivaprakash B., Jagan Mohan R., Shetty P. H. Hyponatraemia secondary to antidepressant therapy: a post marketing safety study. Journal of Pharmacovigilance. 2015;3(3).

| **Supplementary Table 6.** Overview databases per eligible study | |
| --- | --- |
| First author, year | Description of database as mentioned in respective full text |
| Siegler, 1995 | Patient charts of the psychiatry services of the Hospital of the University of Pennsylvania, Philadelphia, from 1988 through 1990 |
| Kirby, 2002 | Patient charts of the psychogeriatric unit of the North-West Hospital in Melbourne between 1997 and 1998 |
| Movig, 2002 | The PHARMO database including information on drug dispensing and hospital admission indications for 320 000 inhabitants of eight Dutch cities, data from 1990 to 1998 were used. |
| Leth-Møller, 2016 | Danish Civil Registration System, the Danish National Patient Register, the Danish register of Medicinal Products Statistics and laboratory data of all individuals born before 1998 and living in The North Denmark Region in the period from 1/01/1998 to 31/12/2012 |
| Rochoy, 2018 | La base nationale française de pharmacovigilance between 01/01/2004 and 31/12/2013 |
| Farmand, 2018 | National Patient Register between 01/10/2005 and 31/12/2014 |
| Mazhar, 2019 | FAERS from 01/2004 to 06/2018 |
| Jun, 2021 | Nationwide population data: Health Insurance Review and Assessment Service (HIRA) Adult Patient Sample database from 2013 to 2017 |
| Mannheimer, 2021 | National Patient Register between 1/10/2006 and 31/12/2014 |
| Huyse, 1994 | Urban university hospital (Free University Hospital, Amsterdam) in The Netherlands, focus on oncology |
| Spigset, 1997 | Pharmacokinetic study by author |
| Fabian, 2003 | Maintenance Therapies in Late Life Depression 2 (MTLD-2) study |
| Fabian, 2004 | University-based ambulatory psychiatric research clinic from 08/1999 to 09/2001 |
| Rowbotham, 2005 | UCSF Pain Clinical Research Center |
| Roxanas, 2007 | First author's clinical practice |
| Shakibaei, 2010 | Sadeghieeh Elderly House and psychiatric clinics of Noor University Hospital, Isfahan |
| Coupland, 2011 | QResearch primary care database: 570 general practices in the United Kingdom |
| Shetty, 2015 | The departments of pharmacology and psychiatry, Mahatma Gandhi Medical College and Research Institute |
| Sarkar, 2021 | The outpatient department of the department of Psychiatry in Burdwan Medical College and Hospital |
| EFFECTS trial, 2020 | Multicentre EFFECTS-trial |
| Brymer, 1992 | Patient charts in Palo Alto Veteran's Affairs Hospital between 01/1989 and 09/1990 |
| Pillans, 1994 | New Zealand Intensive Medicines Monitoring Programme: a national postmarketing event monitoring system, data from 01/1989 to 01/1993 |
| Bouman, 1998 | Patient charts of Department of Health Care of the Elderly, Nottingham from 01/01/1996 to 31/12/1996 |
| Strachan, 1998 | Patient charts of the psychogeriatric inpatient assessment unit from 05/1996 to 04/1997 |
| Wilkinson, 1999 | Patient charts of department of Health Care of the Elderly at the Princess Margaret Hospital, Christchurch |
| Spigset, 1999 | Reports to Swedish Adverse Drug Reactions Advisory Com­mittee |
| Wee, 2004 | Patient charts of sub-acute geriatric centre in Bundoora Extended Care Centre between 01/01/2000 and 31/12/2000 |
| Jung, 2011 | Patient charts of psychiatric inpatient unit at Yeouido St. Mary’s Hospital, the Catholic University of Korea, between 01/01/2002 and 31/12/2009 |
| Montastruc, 2012 | All spontaneous reports of adverse drug reactions registered in 2008 by the Midi-Pyrénées PharmacoVigilance Centre |
| Giorlando, 2013 | Patient charts of public hospital Barwon Health for all psychiatric patients from 01/2006 to 02/2011 |
| Lange-Asschenfeldt, 2013 | Patient charts of Department of Psychiatry and Psychotherapy, University Hospital, Düsseldorf, Germany from 01/01/2004 through 31/12/2006 |
| Noohi, 2016 | Patient charts of medically-ill inpatients assessed by the psychiatric consultation-liaison team of a large Canadian academic hospital between 2008 and 2014 |
| Gandhi, 2017 | Nine linked databases: the Ontario Registered Persons Database, Ontario Drug Benefit Program database, Canadian Institute for Health Infor- mation (CIHI) Discharge Abstract Database, CIHI National Ambulatory Care Reporting System database, Ontario Mental Health Reporting System database, Ontario Health Insurance Plan database, ICES Physician Database and hospital-based serum sodium measurements from Cerner (a medical laboratory service provider) for a subpopulation |
| Albrecht, 2018 | Administrative claims data from US Medicare beneficiaries hospitalized with traumatic brain injury between 2006 and 2010 |
| Grattagliano, 2018 | Databases of 19 general practitioners |
| Revol, 2018 | Base Nationale de Pharmacovigilance (BNPV) |
| Seifert, 2021 | AMSP between 1993 and 2016 |
| Shysh, 2021 | Alberta Health Pharmacy database: patients with a new prescription for citalopram from 2010 until 2017 |
| Nagashima, 2022 | JADER between 01/2004 and 03/2020 |
| FAERS: US Food and Drug Administration (FDA) Adverse Event Reporting System; AMSP: Arzneimittelsicherheit in der Psychiatrie; JADER: Japanese Adverse Drug Event Report; BPNV: La base nationale française de pharmacovigilance | |

# **Supplementary Table 7:** Sensitivity analyses with exclusion of pharmacovigilance studies (primary analyses)

| *Event rates* | k studies | Event rate (95%CI) |  |
| --- | --- | --- | --- |
| Overall antidepressants (any hypoNa) | 28 | 0.0677  (0.0439-0.1031) |  |
| Overall antidepressants (clin hypoNa) | 12 | 0.0160  (0.0057-0.0444) |  |
|  |  |  |  |
| *Crude OR* | k studies | OR (95%CI) | p-value |
| Overall antidepressants (any hypoNa) | 5 | 2.597 (1.538-4.383) | <0.001 |
| Overall antidepressants (clin hypoNa) | 4 | 2.237 (1.258-3.978) | 0.006 |
|  |  |  |  |
| *Adjusted OR* | k studies | OR (95%CI) | p-value |
| Overall antidepressants (any hypoNa) | 5 | 2.410 (1.531-3.794) | <0.001 |
| Overall antidepressants (clin hypoNa) | 3 | 2.879 (0.842-9.853) | 0.092 |

NB: studies excluded from analysis: Rochoy et al.[31]; Mazhar et al.[22], Montastruc et al.[47], Revol et al.[50], Seifert et al.[51], Nagashima et al.[14].

# **Supplementary Figure 1.** Funnel plot and Egger test event rate (any hyponatremia)

White dots indicate observed studies. The black dots indicate imputed data.


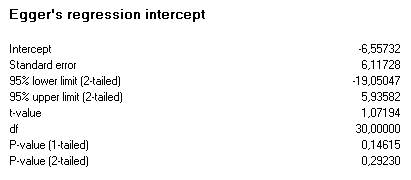


# **Supplementary Figure 2.** Funnel plot odds ratio (any hyponatremia)

Legend: White dots indicate observed studies. The black dots indicate imputed data.

# **Supplementary Figure 3.** Funnel plot head-to-head comparison mirtazapine-SSRI (any hyponatremia)

Legend: White dots indicate observed studies. The black dots indicate imputed data.

# **Supplementary Figure 4.** Funnel plot head-to-head comparison SNRI-SSRI (any hyponatremia)

Legend: White dots indicate observed studies. The black dots indicate imputed data.

# **Supplementary Figure 5.** Funnel plot head-to-head comparison TCA-SSRI (any hyponatremia)

Legend: White dots indicate observed studies. The black dots indicate imputed data.

# **Supplementary Figure 6.** Forest plot event rates overall antidepressants (any hyponatremia)

Legend: Outcome: A. hyponatremia cutoff <135; B. hyponatremia cutoff <130 or clinical case definition

NB. The data of Coupland et al. is based on calculated 1-year adjusted event rate estimates. See Supplementary Table 5. for further comments.

# **Supplementary Figure 7.** Forest plot event rates SSRI (any hyponatremia)

Legend: Outcome: A. hyponatremia cutoff <135; B. hyponatremia cutoff <130 or clinical case definition

NB. The data of Coupland et al. is based on calculated 1-year adjusted event rate estimates. See Supplementary Table 5. for further comments.

# **Supplementary Figure 8.** Forest plot event rates SNRI (any hyponatremia)

Legend: Outcome: A. hyponatremia cutoff <135; B. hyponatremia cutoff <130 or clinical case definition

NB. The data of Coupland et al. is based on calculated 1-year adjusted event rate estimates. See Supplementary Table 5. for further comments.

# **Supplementary Figure 9.** Forest plot event rates TCA (any hyponatremia)

Legend: Outcome: A. hyponatremia cutoff <135; B. hyponatremia cutoff <130 or clinical case definition

NB. The data of Coupland et al. is based on calculated 1-year adjusted event rate estimates. See Supplementary Table 5. for further comments.

# **Supplementary Figure 10.** Forest plot event rates mirtazapine (any hyponatremia)

Legend: Outcome: A. hyponatremia cutoff <135; B. hyponatremia cutoff <130 or clinical case definition

NB. The data of Coupland et al. is based on calculated 1-year adjusted event rate estimates. See Supplementary Table 5. for further comments.

# **Supplementary Figure 11.** Forest plot event rates overall antidepressants (clinically relevant hyponatremia)

Legend: Outcome: A. hyponatremia cutoff <135; B. hyponatremia cutoff <130 or clinical case definition

NB. The data of Coupland et al. is based on calculated 1-year adjusted event rate estimates. See Supplementary Table 5. for further comments.

# **Supplementary Figure 12.** Forest plot event rates SSRIs (clinically relevant hyponatremia)

Legend: Outcome: A. hyponatremia cutoff <135; B. hyponatremia cutoff <130 or clinical case definition

NB. The data of Coupland et al. is based on calculated 1-year adjusted event rate estimates. See Supplementary Table 5. for further comments.

# **Supplementary Figure 13.** Forest plot event rates SNRIs (clinically relevant hyponatremia)

Legend: Outcome: A. hyponatremia cutoff <135; B. hyponatremia cutoff <130 or clinical case definition

NB. The data of Coupland et al. is based on calculated 1-year adjusted event rate estimates. See Supplementary Table 5. for further comments.

# **Supplementary Figure 14.** Forest plot event rates TCAs (clinically relevant hyponatremia)

Legend: Outcome: A. hyponatremia cutoff <135; B. hyponatremia cutoff <130 or clinical case definition

NB. The data of Coupland et al. is based on calculated 1-year adjusted event rate estimates. See Supplementary Table 5. for further comments.

# **Supplementary Figure 15.** Forest plot event rates mirtazapine (clinically relevant hyponatremia)

Legend: Outcome: A. hyponatremia cutoff <135; B. hyponatremia cutoff <130 or clinical case definition

NB. The data of Coupland et al. is based on calculated 1-year adjusted event rate estimates. See Supplementary Table 5. for further comments.

# **Supplementary Figure 16.** Forest plot event rates antidepressant compounds (any hyponatremia)

Legend: Outcome: A. hyponatremia cutoff <135; B. hyponatremia cutoff <130 or clinical case definition

NB. The data of Coupland et al. is based on calculated 1-year adjusted event rate estimates. See Supplementary Table 5. for further comments.

# **Supplementary Figure 17.** Forest plot event rates overall antidepressants for subgroups by cut-off

Legend: Outcome: A. hyponatremia cutoff <135; B. hyponatremia cutoff <130 or clinical case definition

Total between-subgroups effect: Q = 14.31, df = 1, p < 0.001

NB. The data of Coupland et al. is based on calculated 1-year adjusted event rate estimates. See Supplementary Table 5. for further comments.

# **Supplementary Figure 18.** Forest plot event rates SSRIs for subgroups by cut-off

Legend: Outcome: A. hyponatremia cutoff <135; B. hyponatremia cutoff <130 or clinical case definition

Total between-subgroups effect: Q = 22.89, df = 1, p < 0.001

NB. The data of Coupland et al. is based on calculated 1-year adjusted event rate estimates. See Supplementary Table 5. for further comments.

# **Supplementary Figure 19.** Forest plot event rates SNRIs for subgroups by cut-off

Legend: Outcome: A. hyponatremia cutoff <135; B. hyponatremia cutoff <130 or clinical case definition

Total between-subgroups effect: Q = 4.97, df = 1, p = 0.026

NB. The data of Coupland et al. is based on calculated 1-year adjusted event rate estimates. See Supplementary Table 5. for further comments.

# **Supplementary Figure 20.** Forest plot event rates TCAs for subgroups by cut-off

Legend: Outcome: A. hyponatremia cutoff <135; B. hyponatremia cutoff <130 or clinical case definition

Total between-subgroups effect: Q = 5.31, df = 1, p = 0.021

NB. The data of Coupland et al. is based on calculated 1-year adjusted event rate estimates. See Supplementary Table 5. for further comments.

# **Supplementary Figure 21.** Forest plot event rates mirtazapine for subgroups by cut-off

Legend: Outcome: A. hyponatremia cutoff <135; B. hyponatremia cutoff <130 or clinical case definition

Total between-subgroups effect: Q = 5.50, df = 1, p = 0.019

NB. The data of Coupland et al. is based on calculated 1-year adjusted event rate estimates. See Supplementary Table 5. for further comments.

# **Supplementary Figure 22.** Forest plot event rates overall antidepressants (any hyponatremia) for subgroups by age

Legend:
Outcome: A. hyponatremia cutoff <135; B. hyponatremia cutoff <130 or clinical case definition;
Geriatric: N. patient sample does not represent a geriatric cohort; Y. patient sample represents a geriatric cohort

Total between-subgroups effect: Q = 3.53, df = 1, p = 0.060

NB. The data of Coupland et al. is based on calculated 1-year adjusted event rate estimates. See Supplementary Table 5. for further comments.

# **Supplementary Figure 23.** Forest plot event rates SSRIs (any hyponatremia) for subgroups by age

Legend:
Outcome: A. hyponatremia cutoff <135; B. hyponatremia cutoff <130 or clinical case definition;
Geriatric: N. patient sample does not represent a geriatric cohort; Y. patient sample represents a geriatric cohort

Total between-subgroups effect: Q = 0.63, df = 1, p = 0.429

NB. The data of Coupland et al. is based on calculated 1-year adjusted event rate estimates. See Supplementary Table 5. for further comments.

# **Supplementary Figure 24.** Forest plot event rates overall antidepressants (clinically relevant hyponatremia) for subgroups by age

Legend:
Outcome: A. hyponatremia cutoff <135; B. hyponatremia cutoff <130 or clinical case definition;
Geriatric: N. patient sample does not represent a geriatric cohort; Y. patient sample represents a geriatric cohort

Total between-subgroups effect: Q = 2.62, df = 1, p = 0.105

NB. The data of Coupland et al. is based on calculated 1-year adjusted event rate estimates. See Supplementary Table 5. for further comments.

# **Supplementary Figure 25.** Forest plot event rates SNRIs (clinically relevant hyponatremia) for subgroups by age

Legend:
Outcome: A. hyponatremia cutoff <135; B. hyponatremia cutoff <130 or clinical case definition;
Geriatric: N. patient sample does not represent a geriatric cohort; Y. patient sample represents a geriatric cohort

Total between-subgroups effect: Q = 3.32, df = 1, p = 0.068

NB. The data of Coupland et al. is based on calculated 1-year adjusted event rate estimates. See Supplementary Table 5. for further comments.

# **Supplementary Figure 26**. Forest plot crude odds ratios overall antidepressants (any hyponatremia)

Legend:
Outcome: A. hyponatremia cutoff <135; B. hyponatremia cutoff <130 or clinical case definition;
Comparison: X. odds ratios derived from raw data (patients with hyponatremia in exposed and non-exposed groups); Y. crude odds ratios reported in study; Z. adjusted odds ratios reported in study.

# **Supplementary Figure 27**. Forest plot crude odds ratios SSRIs (any hyponatremia)

Legend:
Outcome: A. hyponatremia cutoff <135; B. hyponatremia cutoff <130 or clinical case definition;
Comparison: X. odds ratios derived from raw data (patients with hyponatremia in exposed and non-exposed groups); Y. crude odds ratios reported in study; Z. adjusted odds ratios reported in study.

# **Supplementary Figure 28**. Forest plot crude odds ratios SNRIs (any hyponatremia)

Legend:
Outcome: A. hyponatremia cutoff <135; B. hyponatremia cutoff <130 or clinical case definition;
Comparison: X. odds ratios derived from raw data (patients with hyponatremia in exposed and non-exposed groups); Y. crude odds ratios reported in study; Z. adjusted odds ratios reported in study.

# **Supplementary Figure 29**. Forest plot crude odds ratios TCAs (any hyponatremia)*

Legend:
Outcome: A. hyponatremia cutoff <135; B. hyponatremia cutoff <130 or clinical case definition;
Comparison: X. odds ratios derived from raw data (patients with hyponatremia in exposed and non-exposed groups); Y. crude odds ratios reported in study; Z. adjusted odds ratios reported in study.

* This forest plot pertains to both any and clinically relevant hyponatremia since there were no separate odds ratios for the outcome of <135 mmol available.

# **Supplementary Figure 30**. Forest plot crude odds ratios MAOIs (any hyponatremia)*

Legend:
Outcome: A. hyponatremia cutoff <135; B. hyponatremia cutoff <130 or clinical case definition;
Comparison: X. odds ratios derived from raw data (patients with hyponatremia in exposed and non-exposed groups); Y. crude odds ratios reported in study; Z. adjusted odds ratios reported in study.

* This forest plot pertains to both any and clinically relevant hyponatremia since there were no separate odds ratios for the outcome of <135 mmol available.

# **Supplementary Figure 31**. Forest plot crude odds ratios mirtazapine (any hyponatremia)*

Legend:
Outcome: A. hyponatremia cutoff <135; B. hyponatremia cutoff <130 or clinical case definition;
Comparison: X. odds ratios derived from raw data (patients with hyponatremia in exposed and non-exposed groups); Y. crude odds ratios reported in study; Z. adjusted odds ratios reported in study.

* This forest plot pertains to both any and clinically relevant hyponatremia since there were no separate odds ratios for the outcome of <135 mmol available.

# **Supplementary Figure 32**. Forest plot crude odds ratios overall antidepressants (clinically relevant hyponatremia)

Legend:
Outcome: A. hyponatremia cutoff <135; B. hyponatremia cutoff <130 or clinical case definition;
Comparison: X. odds ratios derived from raw data (patients with hyponatremia in exposed and non-exposed groups); Y. crude odds ratios reported in study; Z. adjusted odds ratios reported in study.

# **Supplementary Figure 33**. Forest plot crude odds ratios SSRI (clinically relevant hyponatremia)

Legend:
Outcome: A. hyponatremia cutoff <135; B. hyponatremia cutoff <130 or clinical case definition;
Comparison: X. odds ratios derived from raw data (patients with hyponatremia in exposed and non-exposed groups); Y. crude odds ratios reported in study; Z. adjusted odds ratios reported in study.

# **Supplementary Figure 34**. Forest plot crude odds ratios SNRI (clinically relevant hyponatremia)

Legend:
Outcome: A. hyponatremia cutoff <135; B. hyponatremia cutoff <130 or clinical case definition;
Comparison: X. odds ratios derived from raw data (patients with hyponatremia in exposed and non-exposed groups); Y. crude odds ratios reported in study; Z. adjusted odds ratios reported in study.

# **Supplementary Figure 35**. Forest plot odds ratios antidepressant compounds (any hyponatremia)

Legend:
Outcome: A. hyponatremia cutoff <135; B. hyponatremia cutoff <130 or clinical case definition;
Comparison: X. odds ratios derived from raw data (patients with hyponatremia in exposed and non-exposed groups); Y. crude odds ratios reported in study; Z. adjusted odds ratios reported in study.

# **Supplementary Figure 36**. Forest plot adjusted odds ratios overall antidepressants (any hyponatremia)

Legend:
Outcome: A. hyponatremia cutoff <135; B. hyponatremia cutoff <130 or clinical case definition;
Comparison: X. odds ratios derived from raw data (patients with hyponatremia in exposed and non-exposed groups); Y. crude odds ratios reported in study; Z. adjusted odds ratios reported in study.

# **Supplementary Figure 37**. Forest plot adjusted odds ratios SSRIs (any hyponatremia)

Legend:
Outcome: A. hyponatremia cutoff <135; B. hyponatremia cutoff <130 or clinical case definition;
Comparison: X. odds ratios derived from raw data (patients with hyponatremia in exposed and non-exposed groups); Y. crude odds ratios reported in study; Z. adjusted odds ratios reported in study.

# **Supplementary Figure 38**. Forest plot adjusted odds ratios SNRIs (any hyponatremia)

Legend:
Outcome: A. hyponatremia cutoff <135; B. hyponatremia cutoff <130 or clinical case definition;
Comparison: X. odds ratios derived from raw data (patients with hyponatremia in exposed and non-exposed groups); Y. crude odds ratios reported in study; Z. adjusted odds ratios reported in study.

# **Supplementary Figure 39**. Forest plot adjusted odds ratios TCAs (any hyponatremia)

Legend:
Outcome: A. hyponatremia cutoff <135; B. hyponatremia cutoff <130 or clinical case definition;
Comparison: X. odds ratios derived from raw data (patients with hyponatremia in exposed and non-exposed groups); Y. crude odds ratios reported in study; Z. adjusted odds ratios reported in study.

# **Supplementary Figure 40**. Forest plot adjusted odds ratios mirtazapine (any hyponatremia)

Legend:
Outcome: A. hyponatremia cutoff <135; B. hyponatremia cutoff <130 or clinical case definition;
Comparison: X. odds ratios derived from raw data (patients with hyponatremia in exposed and non-exposed groups); Y. crude odds ratios reported in study; Z. adjusted odds ratios reported in study.

# **Supplementary Figure 41.** Forest plot odds ratios overall antidepressants (any hyponatremia) for subgroups by age

Legend:
Outcome: A. hyponatremia cutoff <135; B. hyponatremia cutoff <130 or clinical case definition;
Comparison: X. odds ratios derived from raw data (patients with hyponatremia in exposed and non-exposed groups); Y. crude odds ratios reported in study; Z. adjusted odds ratios reported in study.

Geriatric: N. patient sample does not represent a geriatric cohort; Y. patient sample represents a geriatric cohort

Total between-subgroups effect: Q = 0.20, df = 1, p = 0.653

# **Supplementary Figure 42.** Forest plot adjusted odds ratios any antidepressant (any hyponatremia) for subgroups by age

Legend:
Outcome: A. hyponatremia cutoff <135; B. hyponatremia cutoff <130 or clinical case definition;
Comparison: X. odds ratios derived from raw data (patients with hyponatremia in exposed and non-exposed groups); Y. crude odds ratios reported in study; Z. adjusted odds ratios reported in study.

Geriatric: N. patient sample does not represent a geriatric cohort; Y. patient sample represents a geriatric cohort

Total between-subgroups effect: Q = 0.05, df = 1, p = 0.832

# **Supplementary Figure 43.** Forest plot adjusted odds ratios any antidepressant (clinically relevant hyponatremia)

Legend:
Outcome: A. hyponatremia cutoff <135; B. hyponatremia cutoff <130 or clinical case definition;
Comparison: X. odds ratios derived from raw data (patients with hyponatremia in exposed and non-exposed groups); Y. crude odds ratios reported in study; Z. adjusted odds ratios reported in study.

# **Supplementary Figure 44**. Forest plot head-to-head comparison (any hyponatremia)

Legend:
Outcome: A. hyponatremia cutoff <135; B. hyponatremia cutoff <130 or clinical case definition

NB. The incidence data (n and N) for specific antidepressant compounds extracted out of the study by Leth-Møller et al. was only suitable for our head-to-head analyses, see Supplementary Table 5. for further comments.

# **Supplementary Figure 45.** Forest plot head-to-head comparison (clinically relevant hyponatremia)

Legend:
Outcome: A. hyponatremia cutoff <135; B. hyponatremia cutoff <130 or clinical case definition

NB. The incidence data (n and N) for specific antidepressant compounds extracted out of the study by Leth-Møller et al. was only suitable for our head-to-head analyses, see Supplementary Table 5. for further comments.

# **Supplementary Figure 46.** Forest plot head-to-head comparison (any hyponatremia, only geriatric subgroup)

Legend:
Outcome: A. hyponatremia cutoff <135; B. hyponatremia cutoff <130 or clinical case definition

NB. The incidence data (n and N) for specific antidepressant compounds extracted out of the study by Leth-Møller et al. was only suitable for our head-to-head analyses, see Supplementary Table 5. for further comments.

# **Supplementary Figure 47.** Forest plot head-to-head comparison (clinically relevant hyponatremia, only geriatric subgroup)

Legend:
Outcome: A. hyponatremia cutoff <135; B. hyponatremia cutoff <130 or clinical case definition

NB. The incidence data (n and N) for specific antidepressant compounds extracted out of the study by Leth-Møller et al. was only suitable for our head-to-head analyses, see Supplementary Table 5. for further comments.

# **Supplementary Material 1.** Meta-regression event rates overall antidepressants (any hyponatremia)


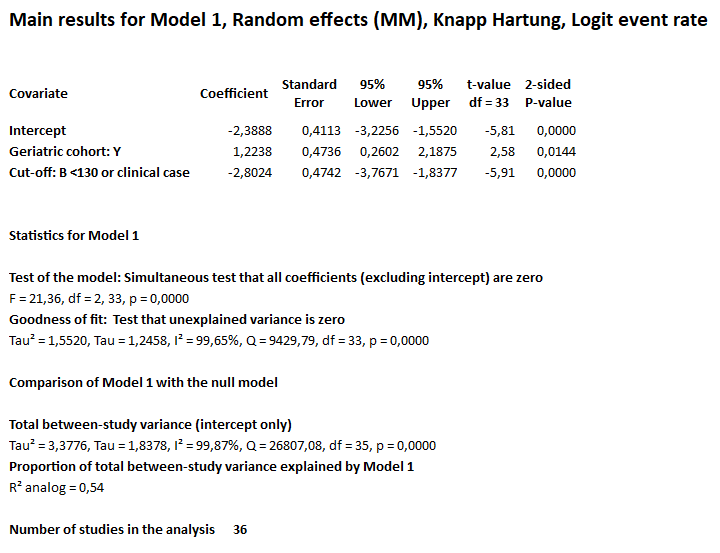


# **Supplementary Material 2:** updates to PROSPERO protocol

**Protocol: Systematic review and meta-analysis of the risk of drug-induced hyponatremia, stratified by antidepressant (class)**Submitted PROSPERO: 29/08/2021; last update: 31/03/2023.
Online versions by date: <https://www.crd.york.ac.uk/prospero/display_record.php?ID=CRD42021269801>

The original protocol was submitted on 29th August 2021, eight updates were performed as listed below. The first literature search ended on 15th October 2022. Before ending the formal screening of search results against eligibility criteria or performing any statistical analysis, we looked for new literature using the same search term and inclusion criteria of the first research on 5th January 2023. This second literature search eventually resulted in 39 studies included in the meta-analysis.

| Version | Original protocol | |
| --- | --- | --- |
| Date | 29 August 2021 | |
| Stage | Preliminary searches | Started |
|  | Piloting of the study selection process |  |
|  | Formal screening of search results against eligibility criteria |  |
|  | Data extraction |  |
|  | Risk of bias (quality) assessment |  |
|  | Data analysis |  |

| Version | First update | |
| --- | --- | --- |
| Date | 05 November 2021 | |
| Comment | The third author was added. | |
| Stage | Preliminary searches | Started |
|  | Piloting of the study selection process |  |
|  | Formal screening of search results against eligibility criteria |  |
|  | Data extraction |  |
|  | Risk of bias (quality) assessment |  |
|  | Data analysis |  |

| Version | Second update | |
| --- | --- | --- |
| Date | 17 December 2022 | |
| Revision note | The anticipated date of completion was changed. | |
| Stage | Preliminary searches | Started and completed |
|  | Piloting of the study selection process | Started and completed |
|  | Formal screening of search results against eligibility criteria | Started |
|  | Data extraction |  |
|  | Risk of bias (quality) assessment |  |
|  | Data analysis |  |

| Version | Third update | |
| --- | --- | --- |
| Date | 18 January 2023 | |
| Revision note | We have added odds ratios for hyponatremia (i.e. any hyponatremia or hypoNa <135mmol/l) and odds ratios clinically relevant hyponatremia (i.e. symptomatic hyponatremia or hypoNa <130mmol/l) to the main outcomes since these are the relevant outcome measures for the case control studies. In hindsight we could have added these before since we have mentioned these odds ratios as one of the 'variables and outcomes' that we would look for in the eventually selected articles. However, we discovered we didn't communicate this clearly and wanted to update this important part of our meta-analysis. | |
| Stage | Preliminary searches | Started and completed |
|  | Piloting of the study selection process | Started and completed |
|  | Formal screening of search results against eligibility criteria | Started |
|  | Data extraction |  |
|  | Risk of bias (quality) assessment |  |
|  | Data analysis |  |

| Version | Fourth update | |
| --- | --- | --- |
| Date | 26 January 2023 | |
| Comment | The anticipated completion day was changed. | |
| Revision note | We need more time to perform our inclusion. | |
| Stage | Preliminary searches | Started and completed |
|  | Piloting of the study selection process | Started and completed |
|  | Formal screening of search results against eligibility criteria | Started |
|  | Data extraction |  |
|  | Risk of bias (quality) assessment |  |
|  | Data analysis |  |

| Version | Fifth update | |
| --- | --- | --- |
| Date | 04 February 2023 | |
| Revision note | We (TG) have screened all hits for the search terms and (TG en FVDE independently) read all full texts of the original articles to see whether they met our inclusion criteria. We have agreed on which articles to include on Thursday 02/02/2023. | |
| Stage | Preliminary searches | Started and completed |
|  | Piloting of the study selection process | Started and completed |
|  | Formal screening of search results against eligibility criteria | Started and completed |
|  | Data extraction | Started |
|  | Risk of bias (quality) assessment |  |
|  | Data analysis |  |

| Version | Sixth update | |
| --- | --- | --- |
| Date | 09 March 2023 | |
| Revision note | After settling on the inclusion of 39 eligible studies we have completed the assessment of study quality and data extraction. Now we have started with the data analysis and are completing this in the next days to weeks. | |
| Stage | Preliminary searches | Started and completed |
|  | Piloting of the study selection process | Started and completed |
|  | Formal screening of search results against eligibility criteria | Started and completed |
|  | Data extraction | Started and completed |
|  | Risk of bias (quality) assessment | Started and completed |
|  | Data analysis | Started |

| Version | Seventh update | |
| --- | --- | --- |
| Date | 30 March 2023 | |
| Revision note | We were able to conclude our meta-analysis. We added a network-meta-analysis to our methodology, which we will present in the supplement. We could not retrieve meta-analysable data about the "time to onset" (weeks before onset of hyponatremia) that we previously mentioned in the record as a secondary outcome and could therefore not report it as an outcome. We mention, however, the onset-time for SSRIs and SNRIs very shortly in the discussion section. The "Additional outcome(s)"-section that was originally submitted can however be seen as "withdrawn" from the record as it did not add anything meaningful to our study. We will submit our manuscript shortly to the journal European Psychiatry (Cambridge Core). | |
| Stage | Preliminary searches | Started and completed |
|  | Piloting of the study selection process | Started and completed |
|  | Formal screening of search results against eligibility criteria | Started and completed |
|  | Data extraction | Started and completed |
|  | Risk of bias (quality) assessment | Started and completed |
|  | Data analysis | Started and completed |

| Version | Eight update | |
| --- | --- | --- |
| Date | 31 March 2023 | |
| Revision note | We focused our meta-analysis on the main research question and made the primary outcome the only outcome (risk for hyponatremia). We deleted the other or secondary outcomes (other risk factors, time to onset of hyponatremia) that we priorly wished to include, albeit for which there was no meta-analyzable data available. | |
| Stage | Preliminary searches | Started and completed |
|  | Piloting of the study selection process | Started and completed |
|  | Formal screening of search results against eligibility criteria | Started and completed |
|  | Data extraction | Started and completed |
|  | Risk of bias (quality) assessment | Started and completed |
|  | Data analysis | Started and completed |

| Version | Ninth update | |
| --- | --- | --- |
| Date | 09 May 2023 | |
| Revision note | The draft manuscript was seen by a professional translator experienced in editing scientific manuscripts. The three authors approved the final wording in the manuscript. We canceled our plan to add a network-meta-analysis (that validated our primary results) in the supplement because of low methodological quality. | |
| Stage | Preliminary searches | Started and completed |
|  | Piloting of the study selection process | Started and completed |
|  | Formal screening of search results against eligibility criteria | Started and completed |
|  | Data extraction | Started and completed |
|  | Risk of bias (quality) assessment | Started and completed |
|  | Data analysis | Started and completed |
